# Reduced connexin-43 expression, slow conduction and repolarisation dispersion in a model of hypertrophic cardiomyopathy

**DOI:** 10.1242/dmm.050407

**Published:** 2024-08-27

**Authors:** Seakcheng Lim, Melissa M. Mangala, Mira Holliday, Henrietta Cserne Szappanos, Samantha Barratt-Ross, Serena Li, Jordan Thorpe, Whitney Liang, Ginell N. Ranpura, Jamie I. Vandenberg, Christopher Semsarian, Adam P. Hill, Livia C. Hool

**Affiliations:** ^1^ Agnes Ginges Centre for Molecular Cardiology at Centenary Institute, The University of Sydney, Sydney 2050, Australia; ^2^ Faculty of Medicine and Health, University of Sydney, Sydney 2050, Australia; ^3^ Victor Chang Cardiac Research Institute, Sydney, 2010, Australia; ^4^ School of Clinical Medicine, Faculty of Medicine and Health, UNSW Sydney, Sydney 2052, Australia; ^5^ Department of Cardiology, Royal Prince Alfred Hospital, Sydney 2050, Australia; ^6^ School of Human Sciences, The University of Western Australia, Crawley 6009, Australia

**Keywords:** Arrhythmia, Cardiovascular disease, Electrophysiology, Stem cells

## Abstract

Hypertrophic cardiomyopathy (HCM) is an inherited heart muscle disease that is characterised by left ventricular wall thickening, cardiomyocyte disarray and fibrosis, and is associated with arrhythmias, heart failure and sudden death. However, it is unclear to what extent the electrophysiological disturbances that lead to sudden death occur secondary to structural changes in the myocardium or as a result of HCM cardiomyocyte electrophysiology. In this study, we used an induced pluripotent stem cell model of the R403Q variant in myosin heavy chain 7 (*MYH7*) to study the electrophysiology of HCM cardiomyocytes in electrically coupled syncytia, revealing significant conduction slowing and increased spatial dispersion of repolarisation – both well-established substrates for arrhythmia. Analysis of rhythmonome protein expression in *MYH7* R403Q cardiomyocytes showed reduced expression of connexin-43 (also known as GJA1), sodium channels and inward rectifier potassium channels – a three-way hit that reduces electrotonic coupling and slows cardiac conduction. Our data represent a previously unreported, biophysical basis for arrhythmia in HCM that is intrinsic to cardiomyocyte electrophysiology. Later in the progression of the disease, these proarrhythmic phenotypes may be accentuated by myocyte disarray and fibrosis to contribute to sudden death.

## Teaser

**Summary:** iPSC-derived cardiomyocytes with the *MYH7* R403Q variant show conduction slowing, increased spatial dispersion of repolarization and reduced expression of some rhythmonome proteins, suggesting a biophysical basis for arrhythmia in hypertrophic cardiomyopathy.

## INTRODUCTION

Hypertrophic cardiomyopathy (HCM) is an autosomal-dominant inherited cardiac disorder with a prevalence of up to one in 200 that can result in arrhythmias, heart failure and sudden death (Ommen et al., 2020; Ommen and Semsarian, 2021). Disease-causing variants associated with HCM most commonly occur in sarcomere genes, including myosin heavy chain 7 (*MYH7*), myosin binding protein C3 (*MYBPC3*) and troponin T (*TNNT2*), with variants in the *MYBPC3* gene being the most common cause (Chiswell et al., 2023). HCM is characterised clinically by left ventricular hypertrophy (wall thickness ≥15 mm) in the absence of loading conditions (Elliott et al., 2014; Writing Committee Members et al., 2020) and is also associated with fibrosis, myocyte disarray and altered energy metabolism (Dhalla et al., 2006). Electrical conduction delays and dispersion of repolarisation, both risk predictors for arrhythmias due to an increased risk of re-entry (King et al., 2013), are also clinical features of HCM (Magrì et al., 2017). However, the correlation between these conduction defects and the histopathology of HCM is limited (Aryana et al., 2007; Wolf et al., 2005; Hueneke et al., 2017). As a result, the mechanisms underlying electrical dysfunction in HCM and its role in sudden death in patients is unclear.

The missense arginine-to-glutamine substitution at position 403 in MYH7 (R403Q) is an HCM variant that causes severe disease, characterised by early-onset and progressive myocardial dysfunction, with a high incidence of sudden cardiac death (Geisterfer-Lowrance et al., 1990; Viola and Hool, 2019). Mice homozygous for this variant (αMHC^403/403^) exhibit neonatal lethality, whereas heterozygous mice (αMHC^403/+^) also demonstrate cardiac dysfunction, myocyte disarray, hypertrophy and fibrosis (Geisterfer-Lowrance et al., 1996). However, consistent with some observations in patient cohorts, neither the extent nor the location of fibrosis correlated with electrical mapping of conduction properties in this mouse model (Wolf et al., 2005). Furthermore, these histopathological changes also did not correlate with the propensity for arrhythmia. It has also been reported that tachyarrhythmias are observed at a far earlier age than the onset of hypertrophy (Hueneke et al., 2017), and that dysfunction of cardiac calcium current and alteration of mitochondrial metabolism occurs prior to the onset of myopathy (Viola and Hool, 2019). Each of these pieces of evidence suggests an alternative pathway for arrhythmic substrate formation in HCM, such as electrical remodelling, that is at least partly independent of alterations in the structure of the myocardium.

In this study, we used a patient-derived induced pluripotent stem cell (iPSC) model of the MYH7 R403Q variant (*MYH7*^403/+^) (Holliday et al., 2018) to investigate whether there are intrinsic electrical properties of HCM cardiomyocyte electrophysiology that provide a biophysical basis for arrhythmia in the absence of structural alteration of the myocardium. Our data show for the first time that in *MYH7*^403/+^ cardiomyocytes, a dramatic reduction in the expression of connexin-43 (also known as GJA1) and sodium channel proteins results in reduced conduction velocity. Accompanying this is an increase in spatial dispersion of repolarisation that establishes potential proarrhythmic substrates and may provide a biophysical basis that contributes to sudden arrhythmic death in patients with HCM.

## RESULTS

### Generation of iPSC lines for *MYH7*^403/+^ and isogenic control

**Fig. 1.**
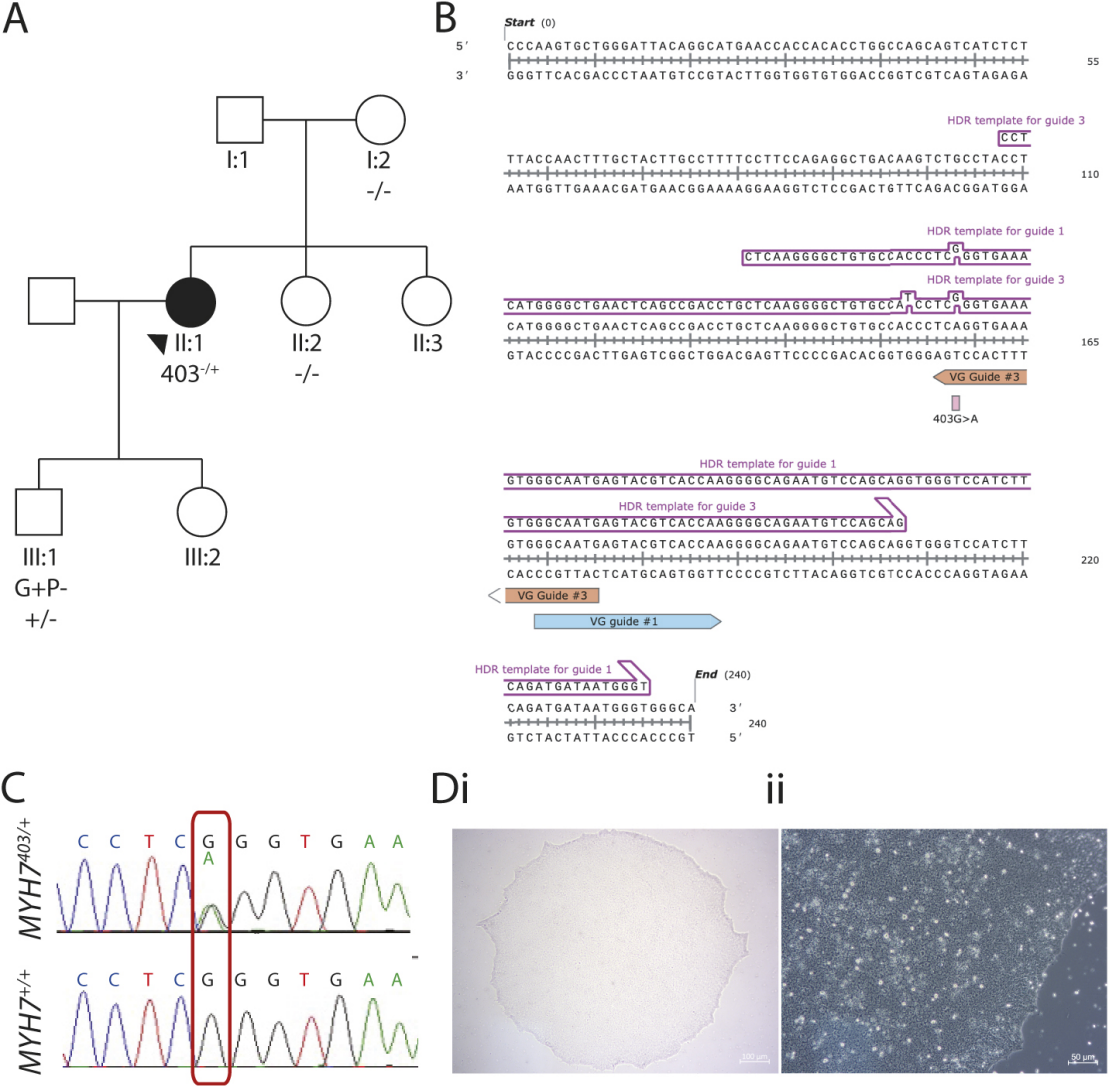
**Generation of isogenic control iPSC line for *MYH7*^403/+^.** (A) *MYH7*^403/+^ induced pluripotent stem cells (iPSCs) were reprogrammed from a patient with hypertrophic cardiomyopathy (II:1) with the *MYH7* p.Arg403Gln variant as previously described (Holliday et al., 2018). (B) Homology-directed repair (HDR) template used to CRISPR-correct the *MYH7* p.Arg403Gln variant in iPSCs. (C) DNA sequencing showing correction of c.1208G>A. (D) Brightfield images of CRISPR-corrected iPSCs at 4× (i) and 10× (ii) magnification. Scale bars: 100 μm (i); 50 μm (ii).

An iPSC line was reprogrammed from a 38-year-old female patient with HCM (II:1, Fig. 1A) with the pathogenic p.Arg403Gln variant in myosin heavy chain 7 (*MYH7*^403/+^) as previously described (Holliday et al., 2018; includes quality control assessment of karyotype, pluripotency and trilineage differentiation capacity). For this study, we generated an isogenic control line by correcting the pathogenic variant in *MYH7* using CRISPR-Cas9 genome editing (*MYH7*^+/+^) (Fig. 1B). Sanger sequencing confirmed the A>G correction at c.1280G>A (Fig. 1C) and showed that no unwanted edits were present in the top ten predicted guide RNA off-target sites. *MYH7*^+/+^ iPSC lines successfully generated colonies of iPSCs with classical morphology of tightly packed cells with a high cell-to-nucleus ratio (Fig. 1D). In all subsequent experiments, this CRISPR-corrected line was used as the comparator for functional and molecular assessments.

### *MYH7*^403/+^ modifies electrophysiology of iPSC-derived cardiomyocytes

**Fig. 2.**
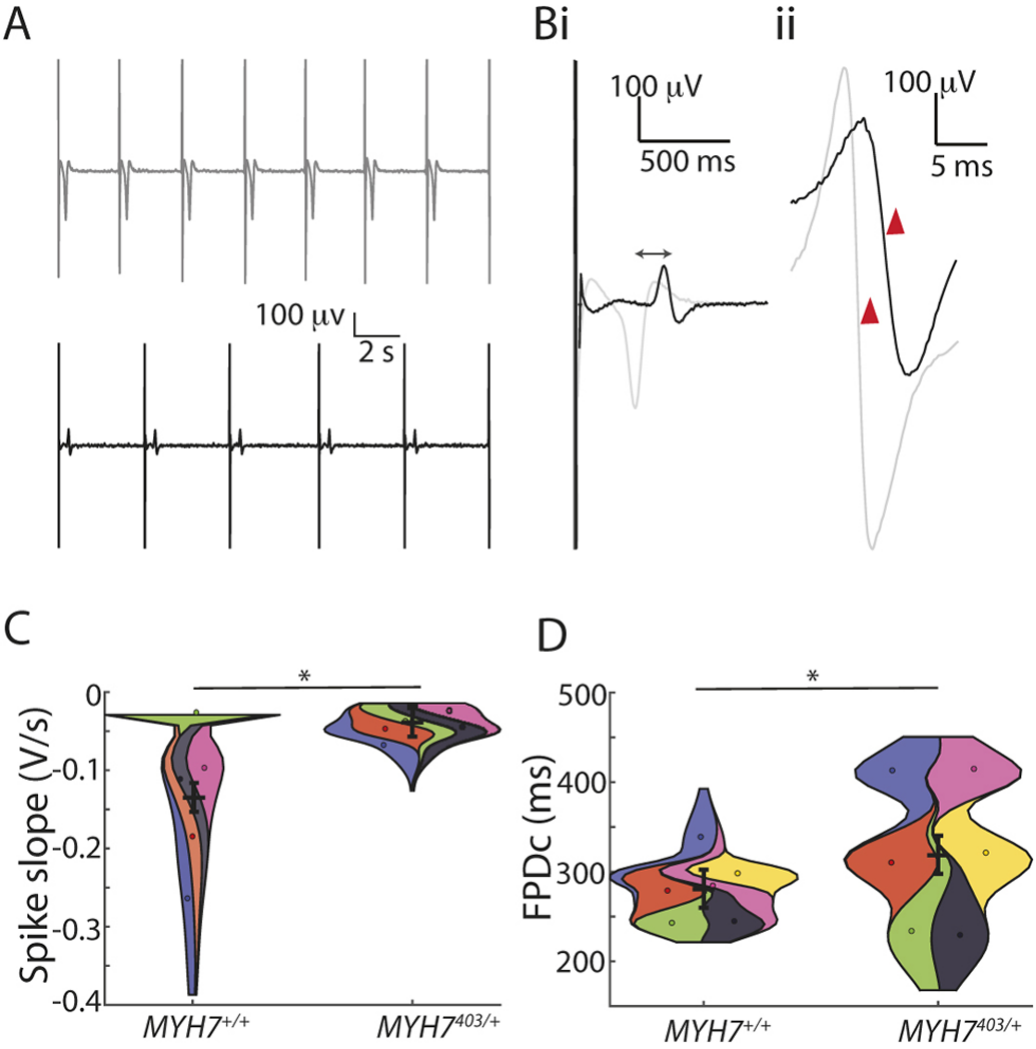
***MYH7*^403/+^ has altered depolarisation and repolarisation in iPSC-CMs.** (A) Representative field potentials from *MYH7*^+/+^ (grey, top) and *MYH7*^403/+^ (black, bottom) iPSC-derived cardiomyocytes (iPSC-CMs). (B) Zoomed view highlighting differences in repolarization time (field potential duration; i) and slope of the depolarization complex (red arrows; ii). (C) Violin SuperPlots summarizing depolarization slope for *MYH7*^+/+^ (*n*=73 replicates/wells from *N*=6 differentiations) and *MYH7*^403/+^ (*n*=89 replicates/wells from *N*=6 differentiations) iPSC-CMs showing significantly slower depolarization for *MYH7*^403/+^ iPSC-CMs (*N*=6, *n*=66 wells). (D) Violin SuperPlots showing rate-corrected field potential duration (FPD_c_) for *MYH7*^+/+^ (*n*=85 replicates/wells from *N*=6 differentiations) and *MYH7*^403/+^ (*n*=111 replicates/wells from *N*=6 differentiations) iPSC-CMs showing significantly prolonged repolarisation for *MYH7*^403/+^ iPSC-CMs. Error bars show mean of *N* ±s.e.m. *P<0.05 (Wald test).

**Fig. 3.**
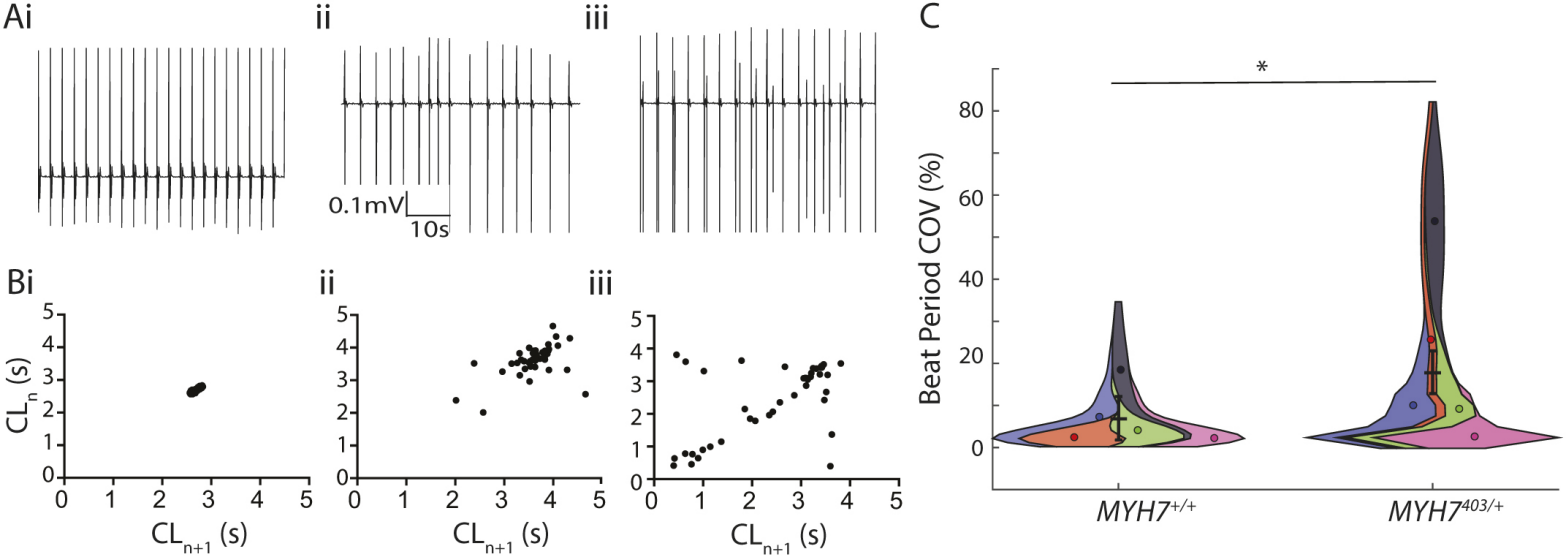
***MYH7*^403/+^ causes irregular/arrhythmic beating in iPSC-CMs.** (A) Example traces of field potentials from *MYH7*^+/+^ (i) and *MYH7*^403/+^ (ii,iii) iPSC-CMs. (B) Poincare plots summarising beat rate irregularity from 2 min recordings in A for *MYH7*^+/+^ (i) and *MYH7*^403/+^ (ii,iii) iPSC-CMs. CL, cycle length. (C) Violin SuperPlot summarising beat period irregularity for *MYH7*^+/+^ (*n*=88 replicates/wells from *N*=6 differentiations) and *MYH7*^403/+^ (*n*=111 replicates/wells from *N*=6 differentiations) iPSC-CMs, showing a significant increase in beat period irregularity for *MYH7*^403/+^ iPSC-CMs. COV, coefficient of variation. Error bars show mean of *N* ±s.e.m. *P<0.05 (Wald test).

Cardiomyocytes derived from iPSCs (iPSC-CMs) for both *MYH7*^403/+^ and *MYH7*^+/+^ were seeded onto microelectrode arrays and extracellular field potentials – *in vitro* surrogates of electrocardiograms – were recorded from spontaneously beating monolayers of cells (Fig. 2A). To rule out any differences in cardiomyocyte purity between *MYH7*^+/+^ and *MYH7*^403/+^ differentiations, flow cytometry was performed to measure ACTN1- and CD90 (also known as THY1)-positive populations, reflecting cardiomyocytes and stromal cells, respectively (Fig. S1). Both *MYH7*^+/+^ and *MYH7*^403/+^ iPSC-CMs showed ∼90% expression of ACTN1 [*MYH7*^+/+^, 95.47±1.05%; *MYH7*^403/+^, 90.40±3.15%; *N*=3 differentiations], with no significant difference between lines. The R403Q variant affected both depolarisation and repolarisation properties of the cardiomyocytes. Specifically, the slope of the depolarisation spike of the field potential (Fig. 2B), a measure of the propagating action potential upstroke, was reduced from −0.14±0.02 V/s in *MYH7*^+/+^ iPSC-CMs to −0.04±0.02 V/s in *MYH7*^403/+^ iPSC-CMs (Fig. 2C), and the Fridericia-corrected field potential duration (FPD_c_; Fig. 2D) was increased from 281.0±21.6 ms in *MYH7*^+/+^ iPSC-CMs to 318±21.5 ms in *MYH7*^403/+^ iPSC-CMs, reflecting slowed repolarisation of the cardiomyocytes (consistent with our previous studies showing prolonged action potential duration in *MYH7*^403/+^ cardiomyocytes; Cserne Szappanos et al., 2023). In addition, *MYH7*^403/+^ iPSC-CMs displayed more irregular/arrhythmic beating (Fig. 3), with a significant increase in the coefficient of variation of beat rate to 17.8±5.2% from 7.0±5.2% in *MYH7*^+/+^ iPSC-CMs (Fig. 3C). For *MYH7*^403/+^ iPSC-CMs, a variety of arrhythmic phenotypes was observed, ranging from more subtle beat-to-beat variability in cycle length (Fig. 3Aii,Bii) to the presence of ectopic depolarisations, often tightly coupled to regular/sinus beats (Fig. 3Aiii,Biii), compared to those seen in *MYH7*^+/+^ iPSC-CMs (Fig. 3Ai,Bi). It should, however, be noted that some ectopic beats observed at slower spontaneous beating rates may be suppressed if monolayers were paced at faster heart rates. β-adrenergic stimulation with 100 nM isoproterenol did not increase beat rate variability for either *MYH7*^+/+^ or *MYH7*^403/+^ iPSC-CMs (Fig. S2).

### Reduced electrical coupling in *MYH7*^403/+^ iPSC-CMs slows conduction and induces spatial dispersion of repolarisation

**Fig. 4.**
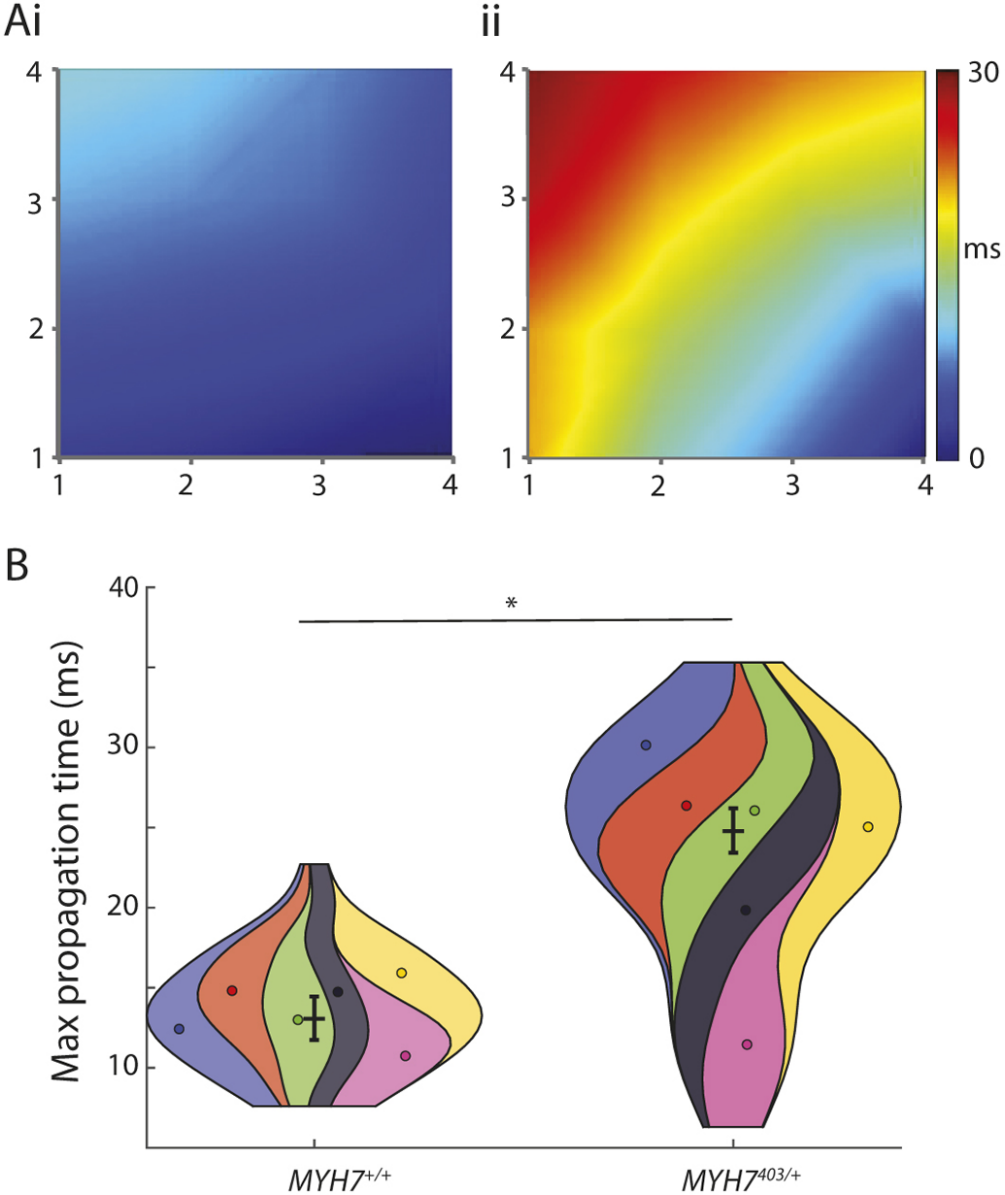
***MYH7*^403/+^ slows conduction velocity in monolayers of iPSC-CMs.** (A) Conduction maps for *MYH7*^+/+^ (i) and *MYH7*^403/+^ (ii) coloured according to activation time measured on a microelectrode array. *X-* and *y*-axis labels represent the electrode position in the array. (B) Violin SuperPlots summarising maximum propagation for *MYH7*^+/+^ (*n*=64 replicates/wells from *N*=6 differentiations) and *MYH7*^403/+^ (*n*=66 replicates/wells from *N*=6 differentiations) iPSC-CMs, showing significantly slower conduction in *MYH7*^403/+^ iPSC-CMs. Error bars show mean of *N* ±s.e.m. *P<0.05 (Wald test).

**Fig. 5.**
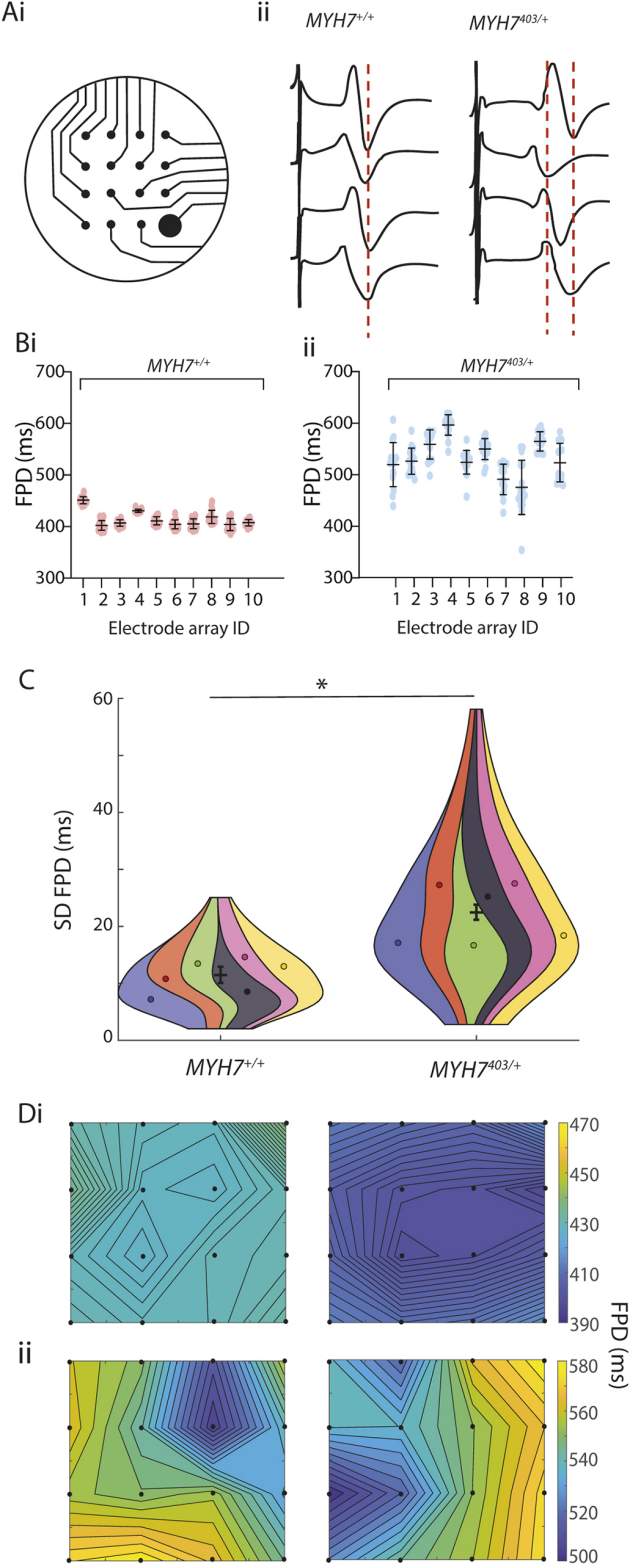
**Increased spatial dispersion of repolarization in *MYH7*^403/+^.** (Ai) Microelectrode array geometry. (Aii) Typical field potentials measured in an individual array for *MYH7*^+/+^ and *MYH7*^403/+^ iPSC-CMs. (B) Range of field potential durations across the 16 electrodes in ten individual arrays for both *MYH7*^+/+^ (i) and *MYH7*^403/+^ (ii) iPSC-CMs. For each array, individual datapoints represent the field potential duration recorded at an individual electrode in the array. Error bars show mean±s.d. (C) Violin SuperPlots summarizing the standard deviation (SD) of field potential duration (FPD) in each electrode array for *MYH7*^+/+^ (*n*=77 wells from *N*=6 differentiations) and *MYH7*^403/+^ (*n*=111 wells from *N*=6 differentiations) iPSC-CMs, showing significantly more dispersion of repolarization times for *MYH7*^403/+^ iPSC-CMs. Error bars show mean of *N* ±s.e.m. *P<0.05 (Wald test). (D) Spatial maps of repolarization duration for *MYH7*^+/+^ (i) and *MYH7*^403/+^ (ii) iPSC-CMs.

A reduced slope of the depolarisation spike (Fig. 2C) might reflect multiple electrophysiological phenomena, including cellular factors, such as a reduced density of sodium currents, or macroscopic electrophysiological properties, such as altered cell–cell coupling, leading to slowed propagation of the activating wavefront. We therefore next measured conduction velocity in monolayers of iPSC-CMs. Conduction maps for *MYH7*^+/+^ and *MYH7*^403/+^ iPSC-CMs, coloured according to activation time measured at individual electrodes across the microelectrode array, are shown in Fig. 4A. In these examples, the activation wavefront takes longer to propagate from the bottom right to the top left of the array in *MYH7*^403/+^ iPSC-CMs (25±1 ms) compared to in *MYH7*^+/+^ iPSC-CMs (13±1 ms) (Fig. 4B), reflecting slower conduction velocity. This slowed conduction velocity suggests that syncytia of *MYH7*^403/+^ iPSC-CMs are less tightly coupled than those of *MYH7*^+/+^ iPSC-CMs. In normal hearts, the level of electric coupling between cells attenuates differences in electrical properties between individual cells. In the context of reduced electrical coupling in *MYH7*^403/+^ iPSC-CMs, we measured the spatial dispersion of repolarization to examine whether intrinsic differences in the repolarization properties of cardiomyocytes in the syncytium are manifested, potentially establishing spatial voltage gradients that might act as a substrate for re-entrant arrhythmia. Field potential durations (FPDs) were measured at each individual electrode in the array (with an interelectrode distance of approximately 300 µm) (Fig. 5Ai), and a greater range of repolarization times was measured in monolayers of *MYH7*^403/+^ iPSC-CMs compared to that in *MYH7*^+/+^ iPSC-CMs (Fig. 5Aii). The spread of FPDs across the 16 electrodes in each array, measured from ten individual electrode arrays each for *MYH7*^403/+^ and *MYH7*^+/+^ iPSC-CMs is shown in Fig. 5B, demonstrating a consistently greater global dispersion of field potentials in *MYH7*^403/+^ monolayers. Overall, the standard deviation of FPDs within each electrode array increased from 11.4±1.4 ms to 22.5±1.3 ms for *MYH7*^+/+^ and *MYH7*^403/+^ iPSC-CMs, respectively (Fig. 5C). Finally, FPDs were mapped relative to the electrode position in the array, showing clear local dispersion of repolarization times of up to 40-50 ms in *MYH7*^403/+^ (Fig. 5Dii) but not *MYH7*^+/+^ iPSC-CMs (Fig. 5Di), potentially resulting in steep voltage gradients that could facilitate re-entrant arrhythmia.

### Molecular basis of electrophysiological phenotype in *MYH7*^403/+^ iPSC-CMs

**Fig. 6.**
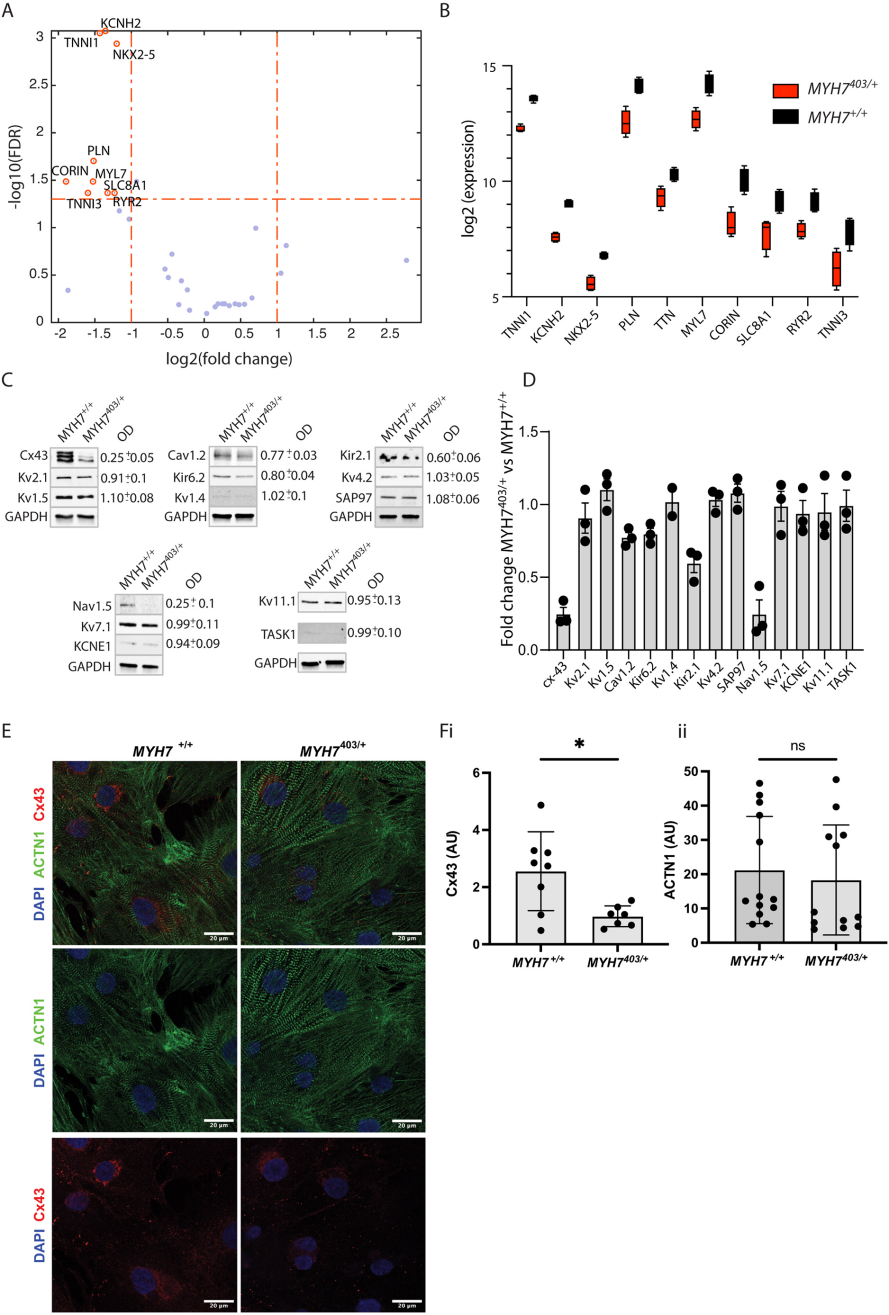
**Molecular basis of electrophysiological changes in *MYH7*^403/+^ iPSC-CMs.** (A) Volcano plot of gene expression changes between *MYH7*^+/+^ and *MYH7*^403/+^ iPSC-CMs. FDR, false discovery rate. (B) Comparison of differentially expressed genes. Boxes show the interquartile range, whiskers show the minimum and maximum, and the median is marked with a line. *N*=4 differentiations. (C) Typical western blots of rhythmonome proteins for *MYH7*^+/+^ and *MYH7*^403/+^. Antibody signals detected on the same membrane are grouped together. Full western blots, including replicates, are provided in Fig. S4. (D) Summary data showing fold change in expression for rhythmonome proteins. *N*=3 biological replicates for all proteins, except K_v_1.4, for which *N*=2. Bars show mean±s.e.m.; exact fold change values measured by optical densitometry (OD) are shown in C. (E) Immunohistochemistry showing expression of connexin-43 (Cx43) and α-actinin (ACTN1) in *MYH7*^+/+^ and *MYH7*^403/+^ cardiomyocytes. (F) Quantification of fluorescence intensity of connexin-43 and α-actinin, normalised to the number of nuclei (*n*=21 images from *N*=3 differentiations). AU, arbitrary units. Error bars show mean±s.d. ns, not significant; *P<0.05 (unpaired two-tailed Student's *t*-test).

To investigate the molecular basis of the observed differences in electrical phenotype, we measured changes in mRNA and protein expression. First, for mRNA, we used a curated panel (nanoString nCounter) targeting cardiac ion channels, calcium-handling proteins and transcription factors. Our data show transcriptional downregulation of rhythmonome genes involved in calcium handling (*PLN*, *RYR2* and *SLC8A1*) and cardiac repolarisation (*KCNH2*); sarcomere genes (*MYL7*, *TTN*, *TNNI1* and *TNNI3*), and genes involved in ventricular cell fate (*NKX2-5* and *CORIN*) (Fig. 6A,B). To complement this, we measured expression of rhythmonome proteins using western blotting (Fig. 6C,D), including connexin-43, Kv2.1 (also known as KCNB1), Kv1.5 (or KCNA5), Cav1.2 (CACNA1C), Kir6.2 (KCNJ11), Kv1.4 (KCNA4), Kir2.1 (KCNJ2), Kv4.2 (KCND2), SAP97 (DLG1), Nav1.5 (SCN5A), Kv7.1 (KCNQ1), KCNE1, Kv11.1 (KCNH2) and TASK1 (KCNK3). Western blotting showed reduced expression of key proteins involved in cardiac excitation and conduction, including 80±12%, 75±5%, and 40±10% reduction in Nav1.5 (the cardiac sodium channel), connexin-43 and Kir2.1 (inward rectifier potassium channel), respectively (Fig. 6D). This reduced expression of connexin-43 observed by western blotting was also confirmed by immunohistochemical analysis, in which fluorescence intensity per cell associated with connexin-43 was reduced 3-fold (Fig. 6E,F).

## DISCUSSION

HCM is an inherited cardiac disorder that results in hypertrophy, fibrosis, myofibre disarray, arrhythmias and sudden cardiac death (Semsarian et al., 2015). Conduction delays and dispersion of repolarisation are clinically associated with HCM (Magrì et al., 2017; Cortez et al., 2017). However, the correlation between conduction defects and the histopathology of HCM is poor, meaning that the mechanism underlying these electrical phenotypes is unclear. In this study, we used an iPSC model of the MYH7 R403Q variant to show that reduced conduction velocity is associated with dramatic reduction in expression of connexin-43 and sodium channel proteins in cardiomyocytes – both key molecular mediators of conduction in the myocardium. Furthermore, we show that this reduced electrical coupling results in significant spatial dispersion of repolarisation – a well-established proarrhythmic substrate (Antzelevitch, 2007; Zahid et al., 2016) – which may provide a biophysical basis that contributes to sudden arrhythmic death in patients with HCM.

### *MYH7*^403/+^ slows conduction in iPSC-CM monolayers

HCM is a disease that has been modelled in a range of systems including intact heart muscle strips, isolated cardiomyocytes, myofibrils, purified actomyosin and animal models (Mosqueira et al., 2019). More recently, iPSC-CM models of HCM related to variants in *MYH7* (Lan et al., 2013; Dainis et al., 2020) as well as other genes (Cohn et al., 2019; Wu et al., 2019) have been shown to reproduce key elements of the HCM phenotype *in vitro*, including cellular morphology and/or hypertrophy (Cohn et al., 2019), elevated metabolism (Toepfer et al., 2019), disrupted calcium handling and hypercontractility (Lan et al., 2013; Wu et al., 2019). In relation to electrophysiology, data from iPSC models are less extensive and inconsistent. Studies of *MYH7-*related HCM models in iPSC-CMs report variable electrical phenotypes, with both unchanged or prolonged action potential duration reported, as well as irregular beating (Lan et al., 2013; Han et al., 2014). However, there are no *in vitro* studies using iPSC models that examine macroscopic electrophysiological properties that might provide a biophysical explanation for the conduction abnormalities seen in patients.

In this study, we used microelectrode arrays to record electrical signals from monolayers of iPSC-CMs, allowing us to study properties such as conduction velocity and repolarisation dispersion. Our data showed, for the first time, significantly slowed conduction velocity (approximately 50% reduction; Fig. 4) in an *in vitro* model of HCM. Reduced conduction velocity increases the chance of arrhythmia by reducing the spatial scale over which re-entry is possible (Janse and Wit, 1989). In the clinical setting, slow conduction has been reported in patients with HCM (Schumacher et al., 2005; Saumarez, 1994). Given the histopathology of the HCM myocardium, particularly regarding the presence of interstitial fibrosis, it is generally assumed that disruption of normal electrical propagation is a function of this fibrosis and the associated reduction in coupling between cardiomyocytes (Lyon et al., 2018; Varnava et al., 2001). However, several studies have suggested that this is not necessarily always the case. For example, in the clinical setting, Aryana et al. (2007) reported a lack of concordance between imaging of intramural fibrosis and low voltage on endocardial or epicardial mapping. Similarly, in αMHC^403/+^ mice, Wolf et al. (2005) demonstrated that neither the extent nor the location of fibrosis correlated with electrical mapping of conduction properties, nor did this correlate with the propensity for arrhythmia. Furthermore, in a separate mouse study, Hueneke et al. (2017) showed that tachyarrhythmias are observed at a far earlier age than the onset of hypertrophy, suggesting an alternative pathway for arrhythmogenesis in HCM that is independent of alterations to the structure of the myocardium. Our data are consistent with these observations and support a biophysical basis for conduction slowing in *MYH7*^403/+^ iPSC-CMs intrinsic to cardiomyocyte electrophysiology, which may be accentuated by myocyte disarray in addition to fibrosis and later in the progression of disease.

### Reduced electrical coupling between *MYH7*^403/+^ iPSC-CMs results in increased spatial dispersion of repolarisation

Spatial dispersion or regional differences in repolarisation times facilitate the initiation and maintenance of re-entrant arrhythmias (Mines, 1913). This view is supported by studies showing that regional differences in repolarisation time lead to an increased susceptibility to arrhythmias in response to premature stimulation (Han and Moe, 1964; Clayton and Holden, 2005; Boersma et al., 2002). Similarly, previous work has shown a tight correlation between the range and variability in action potential duration across the tissue and the duration of the vulnerable window for initiation of re-entry (Clayton and Holden, 2005), whereas on patient electrocardiograms, measurements of T wave area, a metric that reflects dispersion of repolarisation, are predictive of ventricular arrhythmias (Nearing et al., 2012). Such spatial dispersion of repolarisation is thought to arise because of variation in ion channel gene expression or ion channel function in different regions of the heart. However, in normal healthy tissue, tight electrotonic coupling ensures that repolarisation of individual cells or different regions of the tissue is synchronised and, hence, is less vulnerable to arrhythmia (Lesh et al., 2018; Joyner, 1986).

In our iPSC model of MYH7 R403Q, we hypothesised that the observed reduction in conduction velocity reflected reduced electrical coupling, and that this might alter the degree to which repolarisation was synchronised between cells, manifesting in increased spatial dispersion of repolarisation. This was indeed the case, with significantly greater variability and range of FPDs (reflective of repolarisation time) measured across *MYH7*^403/+^ monolayers compared to their CRISPR-corrected controls (Fig. 5). Moreover, these voltage gradients occurred over small, local spatial scales. Differences of up to 150 ms in FPDs were recorded across electrode arrays spaced over areas just 1-2 mm across, with steep voltage gradients occurring even between neighbouring electrodes (see example in Fig. 5Dii). In relation to arrhythmogenesis, this is important as it is thought that local repolarisation dispersion (as opposed to global differences) often serves as a source for the ectopic beats that trigger arrhythmias (Dunnink et al., 2015; Tan et al., 2017). Such dispersion of repolarisation has been linked to arrhythmogenesis in a range of inherited and acquired disorders including long QT syndrome (Antzelevitch, 2007; Lachaud et al., 2022), Brugada syndrome (Antzelevitch, 2007), heart failure (McIntosh et al., 2000) and post-ischaemia (Gough et al., 2018), in which voltage gradients occur variously due to factors including regional differences in ion channel expression as well as electrical uncoupling following gap junction downregulation. Specifically, in relation to HCM, Saumarez et al. (2018) reported fractionated conduction related to myocardial disarray and fibrosis on patient electrograms, whereas Hueneke et al. (2017) reported dispersion of repolarisation on a global scale, related to differential expression of potassium channel genes in different regions of the mouse heart. Our observations of highly discordant local repolarisation in *MYH7*^403/+^ monolayers are therefore consistent with a mechanism that could contribute to arrhythmic susceptibility in patients. To our knowledge, this is the first report of such pronounced local spatial dispersion of repolarisation in a model of HCM that results from the biophysical properties of the HCM cardiomyocyte.

### Molecular basis of electrophysiological changes in *MYH7*^403/+^ iPSC-CMs

Conduction of electrical signals in the ventricular myocardium is regulated primarily by voltage-gated sodium channels (e.g. Nav1.5), which are the main contributors to cardiomyocyte depolarisation, and gap junctions (primarily connexin-43 in the ventricle) that allow the flow of current between coupled cells. Loss of either of these critical contributors to cardiac conduction can disrupt regular electrical propagation and increase the risk of arrhythmias. Our analysis of protein expression showed a dramatic (∼80%) reduction in the expression of both connexin-43 and Nav1.5 proteins in *MYH7*^403/+^ cardiomyocytes compared to that in their CRISPR-corrected controls. In addition to this, we also measured a ∼50% reduction in the levels of the Kir2.1 protein (an inward rectifier potassium channel). Functionally, this reduction in Kir2.1 levels would serve to depolarize the resting membrane potential, leading to functional inactivation of the remaining Nav1.5 population, thus acting as a potential ‘third hit’ on electrical conduction. Although we cannot be certain as to why a pathological variant in *MYH7* might lead to these electrophysiological changes, one potential candidate is an effect of altered calcium cycling in *MYH7*^403/+^ cardiomyocytes on signalling pathways and gene expression, called excitation transcription coupling (Dewenter et al., 2017). Previous studies of this variant in mice (Semsarian et al., 2002) have reported altered expression of calcium-handling proteins, which was corrected by pharmacotherapy targeted at normalisation of calcium handling, pointing to a role of calcium homeostasis in regulation of gene expression in this *MYH7* variant. In a similar manner, it is possible that abnormal calcium handling also contributes to reduced expression of connexins and the sodium channels observed here.

The degree of reduction in conduction velocity observed (Fig. 4) is consistent with previous studies that have examined a similar loss of connexin expression in other systems. In a mouse model of conditional deletion of connexin-43, van Rijen et al. (2004) showed that a 70-95% conditional deletion of connexin-43 was necessary to reduce conduction velocity or increase the dispersion of conduction, whereas a 50% loss of function had no effect. Similarly, an earlier study by Reaume et al. (1995) showed conduction slowing of between 42% and 56% associated with a 95% reduction in connexin-43 protein expression. As a result of these observations, although reduced expression and/or localisation of connexin-43 has been observed in several pathological states, including post-infarction (Zhang et al., 2009) and in HCM (Kostin et al., 2004), it has been considered unlikely that the degree of reduced expression reported for these diseases was sufficient to directly affect conduction and hence promote arrhythmogenesis. Rather, a second factor such as fibrosis or collagen deposition was likely responsible for the increased propensity for arrhythmias in these patients. The mechanism of conduction slowing reported here for *MYH7*^403/+^ cardiomyocytes is therefore unique in that it is the first time that reduced expression of connexin protein has been measured in a model of an HCM gene variant of a sufficient magnitude to directly impact cardiac conduction. Moreover, this effect is likely amplified by a simultaneous reduction in the expression of sodium channels and inward rectifier channel protein in a triumvirate of altered rhythmonome protein expression that coalesce to slow cardiac conduction.

### Transcriptomic analysis of *MYH7*^403/+^ iPSC-CMs

Transcriptome data showed that genes related to calcium handling, cellular electrical repolarisation, sarcomere structure and cardiac cell fate are differentially expressed between *MYH7*^+/+^ and *MYH7*^403/+^ cardiomyocytes. First, expression of *NKX2-5* was 2.3-fold higher in normal cardiomyocytes compared to that in *MYH7*^403/+^ cardiomyocytes. NKX2-5 is a key transcription factor involved in cardiac development and cell fate across species (Elliott et al., 2010). Of relevance to this study, NKX2-5 dysregulation is associated with defects in cardiac conduction and electrophysiology (Ellesøe et al., 2016), including reduced expression of connexins (Nakashima et al., 2009; Briggs et al., 2008) as well as Nav1.5, RyR2 and Kv11.1 (Briggs et al., 2008), consistent with our transcriptomic and protein data (Fig. 6). Similarly, a mouse model employing an inducible system for disruption of notch signalling, which resulted in downregulation of NKX2-5, displayed a strikingly similar electrical phenotype to that seen here, including slow conduction velocity and irregular beating related to reduced expression of gap junctions and sodium channels (Qiao et al., 2017). Another gene that was downregulated, which is a target for NKX2-5, was *CORIN*. Corin is a serine protease highly expressed in the heart that has conserved binding sequences for NKX2.5 in its 5′ flanking regions (Pan et al., 2002). Corin has previously been identified as a cell surface marker for ventricular cell populations (Zhang et al., 2019) and has been reported as being dysregulated (both downregulated and upregulated) in hypertrophy and heart failure (Khoury et al., 2021; Tran et al., 2004; Ichiki et al., 2013; Langenickel et al., 2004). Furthermore, reactome pathway analysis also associates corin with cardiac conduction (Fabregat et al., 2018). Taken together with the altered expression of NKX2-5 in this model of *MYH7*^403/+^ cardiomyocytes, our transcriptome data are therefore consistent with the *in vitro* electrical phenotypes reported here. Furthermore, if maintained during development and into adulthood in patients, these changes would likely contribute to increased risk of arrhythmias and sudden death.

In relation to calcium handling, we observed downregulation of a cluster of genes including *RYR2* (encoding the ryanodine receptor), *SLC8A1* (encoding the sodium-calcium exchanger) and *PLN* (encoding phospholamban). A wealth of previous studies has reported altered calcium homeostasis in models of HCM (Viola and Hool, 2019; Lan et al., 2013; Robinson et al., 2018; Helms et al., 2016; Semsarian et al., 2001) and indeed have identified disrupted calcium handling as central to driving HCM pathology (Lan et al., 2013; Semsarian et al., 2001). In particular, there was altered expression of key calcium-handling proteins, including RyR2 (Semsarian et al., 2001), in agreement with the transcriptome data we report here. The third cluster of genes downregulated in *MYH7*^403/+^ cardiomyocytes was related to sarcomere structure – specifically troponin I (both *TNNI1* and *TNNI*3 isoforms), myosin light chain 7 (*MYL7*) and titin (*TNN*). Although there were no changes in *TNNI1:TNNI3* or *MYL2:MYL7* ratios, that might reflect a less mature or less ventricular cell identity of *MYH7*^403/+^ cardiomyocytes (Fig. S3), and the broad downregulation of sarcomere genes observed here might reflect overall reduced structure and order of the sarcomere associated with this variant in a thick filament protein (myosin heavy chain).

Finally, related to cardiac repolarisation, expression of the *KCNH2* gene, encoding the pore-forming subunit Kv11.1 of the rapid delayed rectifier potassium current, one of the main drivers of action potential repolarisation in human ventricles, was reduced. This is consistent with studies in mice, from our group and others, that have shown downregulation of potassium channel gene expression associated with HCM variants in both *MYH7* (Hueneke et al., 2017; Cserne Szappanos et al., 2023) and *MYBPC3* (Flenner et al., 2021). However, in these studies, a reduction in protein expression or ion channel current density was also reported in association with the reduced gene expression. Conversely, we saw no significant change in the protein levels of the ion channel Kv11.1 encoded by this gene in *MYH7*^403/+^ versus CRISPR-corrected controls (as well as only modest changes in repolarisation time). Similar results were also reported by Flenner et al. (2021), where data from either human iPSCs or from human ventricular samples showed no reduction in K^+^ currents, in contrast to their mouse data. Such discordance between mRNA and protein levels is common (Liu et al., 2016), with specific examples reported regarding potassium channels and arrhythmias (Brundel et al., 2001). This most likely reflects that protein abundance is in large part controlled at the post-transcriptional and translational levels, and that these processes can adapt to compensate for changes in mRNA transcription to keep cellular protein levels at appropriate levels for normal function.

A similar analysis of transcriptome changes associated with the same R403Q variant was previously undertaken by Cohn et al. (2019) using RNA sequencing. Although the methods used in their study (RNA sequencing) compared to ours (nanoString) are not directly comparable, and a higher threshold for false discovery was used here, there are similarities between the results. For example, some of the genes discussed above associated with calcium handling (*PLN*) and sarcomere structure (*MYL7*) were differentially expressed in both studies. Similarly, expression of the *SCN5A* gene, encoding the cardiac sodium channel Nav1.5 was reduced in Cohn et al. (2019). Although this gene did not reach the threshold for significance for differential gene expression in our study, we do report lowered expression of the Nav1.5 protein here, which we propose, together with altered expression of connexin-43, to be the driver of the reduced conduction velocity reported in this study.

### Conclusions

In this study, we have shown that the R403Q pathogenic variant in *MYH7*, a common and severe cause of HCM, results in changes to cardiomyocyte electrophysiology that may contribute to proarrhythmia and increased risk of sudden death in patients. Specifically, in monolayers of *MYH7*^403/+^cardiomyocytes, reduced electrical coupling resulted in significantly slowed conduction velocity and an accompanying increase in spatial dispersion of repolarization that established steep voltage gradients in monolayers of iPSC-derived ‘pseudo-tissue’. Analysis of rhythmonome proteins revealed that reduced electrical coupling resulted from lowered expression of key proteins – specifically connexin-43, Nav1.5 and Kir2.1 – that support electrical conduction between cardiac cells. This is the first report of a cardiomyocyte-intrinsic mechanism of disrupted electrical coupling and conduction in a model of HCM that may represent a new focus for targeted antiarrhythmics in patients with HCM. Furthermore, this biophysical basis for proarrhythmia in HCM may also be accentuated by structural changes in the myocardium later in the progression of disease to contribute to sudden arrhythmic death in these patients.

#### Limitations

Although we observed proarrhythmic electrical phenotypes (slow conduction, irregular beating and increased dispersion of repolarisation) in our iPSC model of R403Q, we did not observe overt re-entrant activity in our experiments. A likely explanation for this is the small spatial scale of the monolayers studied (microelectrode array geometry is on the order of 2 mm) that, even in the context of slow conduction and discordant repolarisation, was not sufficiently large to support re-entry. It may also be the case that the changes in the expression of rhythmonome proteins observed here and their associated phenotypes may be accentuated by the relatively immature properties of iPSC-CMs and may be less prominent in more mature cells. In our study, we used one iPSC clone to model the effects of the R403Q variant; if performed on multiple clones, there may have been a difference in arrhythmia vulnerability. Finally, data from our study were acquired from one clone from a single patient (and its CRISPR-corrected isogenic control). Further studies including additional clones from cell lines from multiple patients with the same variant in *MYH7* would add further strength to our conclusions and help quantify the role of the genetic background in fine-tuning the emergent electrical phenotype of the R403Q variant.

## MATERIALS AND METHODS

### Generation of patient-specific human iPSCs and general cell culture maintenance

Human iPSCs were derived from a patient carrying the HCM-causing variant p.Arg403Gln in *MYH7*. Peripheral blood mononuclear cells were isolated and reprogrammed iPSCs were generated as previously described (Holliday et al., 2018). iPSC lines were routinely screened for mycoplasma infection every 12 months. Patient informed consent, use and generation of patient-derived iPSCs complied with national guidelines with oversight by the Sydney Local Health District Committee (protocol X19-0108 and ETH00461). iPSC colonies were maintained in a defined, feeder cell-free medium, mTeSR1 PLUS (StemCell Technologies, BC, Canada) and the extracellular matrix, Matrigel hESC-qualified matrix (Corning, NY, USA), and passaged as aggregates using ReLeSR passaging reagent (StemCell Technologies). Brightfield images were captured on the Zeiss Primo Vert inverted microscope and processed on the Zeiss ZEN Lite 3.4 software (Zeiss, Baden-Württemberg, Germany).

### CRISPR-Cas9 gene editing of iPSCs

The guide RNAs (5ʹ-CATTGCCCACTTTCACCTGA-3ʹ) for generation of isogenic control lines were designed using a gRNA design tool (Massachusetts Institute of Technology, MA, USA) and ordered as oligonucleotides to be cloned into pSpCas9(BB)-2A-Puro (PX459) V2.0 (Addgene, MA, USA; #62988). Homology-directed repair donor oligonucleotides (5ʹ-C*C*TCATGGGGCTGAACTCAGCCGACCTGCTCAAGGGGCTGTGCCATCCTCGGGTGAAAGTGGGCAATGAGTACGTCACCAAGGGGCAGAATGTCCAGC*A*G-3ʹ) were designed to flank the Cas9 cut site and ordered as ultramers that contained two phosphorothioate bonds on each end of the oligonucleotide (asterisks) (Integrated DNA Technologies, IA, USA).

iPSCs were plated as single cells and transfected 24 h later with 1 µg plasmid and 5 µl of 100 µM donor oligonucleotide using Lipofectamine Stem (Thermo Fisher Scientific, MA, USA) according to the manufacturer's protocol. After transfection for 24 h, cells were selected with 0.5 µg/ml puromycin for a further 24 h. Single cells were selected into a 96-well plate for Sanger sequencing to determine successfully edited clones, which were further expanded into established cell lines. Off-target analysis involved Sanger sequencing of ten potential guide RNA off-target sites (http://www.rgenome.net/cas-offinder/), Sanger sequencing of all *TP53* exons and molecular karyotyping (Victorian Clinical Genetics Services, VIC, Australia) to ensure genomic integrity.

### Cardiomyocyte differentiation

Cultures of iPSCs at 70-80% confluency were dissociated into single-cell suspensions with TryPLE (Thermo Fisher Scientific) for 7 min at 37°C and 5% CO_2_. Cells were then plated between 450,000 and 750,000 cells per well of a Matrigel-coated 12-well tissue culture plate in mTeSR1 PLUS supplemented with 10 µM ROCK inhibitor (Y-27632) (Reprocell, MD, USA) and differentiated into cardiomyocytes using the StemDiff Ventricular Cardiomyocyte Differentiation Kit (StemCell Technologies) as per the manufacturer's protocol. For downstream experiments, iPSC-CMs that showed consistent beating across the entire culture area were used.

### Multi-electrode array recordings

At day 15 (±2 days) of differentiation, beating iPSC-CMs were dissociated using a modification of the two-step collagenase/trypsin protocol from Mills et al. (2017). Briefly, iPSC-CMs were incubated with 0.2% collagenase type I (Thermo Fisher Scientific) in PBS supplemented with 20% fetal bovine serum (Cytiva) for 45 min at 37°C and 5% CO_2_, then centrifuged at 300 ***g*** for 3 min. iPSC-CMs were incubated in 0.25% trypsin-EDTA (Thermo Fisher Scientific) for 10 min at room temperature, then filtered through a 40 µm cell strainer. iPSC-CMs were plated at a density of 80,000 cells/well of an Axion Biosystems E-Stim+ Classic Multi-electrode Array (MEA) 48-well plate (Axion Biosystems, GA, USA), and field potentials and conduction velocities were recorded on days 30-35 using the Maestro-APEX MEA system (Axion Biosystems). iPSC-CMs were maintained in α-MEM (Thermo Fisher Scientific) supplemented with 200 µM L-ascorbic acid 2-phosphate sesquimagnesium salt hydrate (Sigma-Aldrich) and 2% B27+insulin (Thermo Fisher Scientific) until day 45 post differentiation or final collection for downstream assays. For FPDs, repolarisation time was measured from the point of maximum slope of the depolarization spike to the peak of the repolarizing ‘T wave’. Spontaneous activities of iPSC-CMs were recorded at 37°C and 5% CO_2_. Acquisition and analysis were performed using AxIS v2.5.1.10 software (Axion Biosystems), the Cardiac Analysis Tool (Axion Biosystems), in-house MEA analysis software (Victor Chang Cardiac Research Institute, NSW, Australia) and MATLAB R2021a (MathWorks, MA, USA). For rate correction of FPDs, the Fridericia formula was applied, according to the equation below:

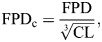
where ‘FPD_c_’ is the rate-corrected FPD in milliseconds, ‘FPD’ is the measured FPD in milliseconds and CL is the cycle length in seconds.

### Western blotting

At day 45 (±2 days) post differentiation, protein was extracted from beating iPSC-CMs in protein lysis buffer [1× Pierce RIPA buffer (Thermo Fisher Scientific), 1× PhosSTOP cocktail (Sigma-Aldrich), 1× cOmplete protease inhibitor (Sigma-Aldrich)]. Protein concentration was quantified using the Bradford assay using bovine serum albumin (BSA) as a standard. 25 µg of total protein was loaded into precast 10% Mini-PROTEAN TGX Stain-Free SDS-polyacrylamide gels (Bio-Rad), then electrophoretically transferred to 0.2 µm nitrocellulose membrane (Trans-Blot Turbo Transfer Pack, Bio-Rad) using the Bio-Rad Trans-Blot Turbo Transfer System. Blots were probed with the following primary antibodies: rabbit polyclonal anti-Kv1.4 (Alomone, APC-167, 1:500), rabbit polyclonal anti-Kv1.5 (Alomone, APC-004, 1:500), guinea pig polyclonal anti-Kv2.1 (Alomone, AGP-109, 1:1000), rabbit polyclonal anti-Kv4.2 (Alomone, APC-023, 1:500), guinea pig polyclonal anti-Kir2.1 (Alomone, AGP-044, 1:500), rabbit polyclonal anti-K2P3.1 (TASK1) (Alomone, APC-024, 1:500), rabbit polyclonal anti-Kir6.2 (Alomone, APC-020, 1:500), rabbit polyclonal anti-Kv11.1 (HERG) (Alomone, APC-109-F, 1:500), rabbit polyclonal anti-KCNE1 (IsK, MinK) (Alomone, APC-163, 1:500), rabbit polyclonal anti-KCNQ1 (Kv7.1) (Alomone, APC-168, 1:500), rabbit polyclonal anti-SAP97 (Thermo Fisher Scientific, PA1-741, 1:1000), rabbit polyclonal anti-Nav1.5 (Alomone, ASC-005, 1:500), rabbit polyclonal anti-Cav1.2 (Alomone, ACC-003, 1:500), rabbit polyclonal anti-connexin 43 (Cell Signalling Technology, 3512, 1:1000) and rabbit monoclonal anti-GAPDH (Cell Signalling Technology, #2118, 1:2000). Blots were subsequently with the pre-absorbed, polyclonal goat anti-rabbit IgG (H&L) HRP (Abcam, ab97040, 1:10,000) or polyclonal goat anti-guinea pig IgG (H&L) HRP (Abcam, ab97155, 1:10,000) secondary antibody. Whenever it was possible, we probed the membranes in a sequential manner with antibodies from different origins. When blots were used for re-probing with multiple antibodies from the same origin, blots were stripped between probing using stripping buffer (consisting of 62.5 mM Tris HCl, pH 6.8, 100 mM 2-mercaptoethanol and 2% SDS) for 30 min at 50°C. Chemiluminescence signal was detected after incubation of the blots with Immobilon Crescendo Western HRP substrate detection reagent (Millipore, WBLUR0500) following the manufacturer's instructions, by using the Bio-Rad ChemiDoc MP Imaging System. Densitometry was performed for each antibody using ImageJ software (Schneider et al., 2012). Background-subtracted intensity values were normalized to loading control GAPDH signal intensity detected on the same blot. All immunoblot experiments were run as triplicate. Representative images are shown in Fig. 6C and uncropped blots are provided in Fig. S4.

### nanoString analysis

RNA was harvested with 100 µl of QIAzol lysis reagent (QIAGEN) per well of an MEA plate at the completion of functional assays at ∼day 45. RNA was pooled from three to four wells of an MEA plate and then extracted using the miRNeasy kit (QIAGEN). RNA levels were measured using the nanoString nCounter PlexSet (nanoString, WA, USA) according to the manufacturer's instructions and gene expression analysed using nSolver software (nanoString). A two-stage Benjamini, Krieger and Yekutieli procedure was implemented for controlling the false discovery rate (Benjamini et al., 2006). A false discovery rate of 5% and a fold change of 2 in gene expression were considered significant.

### Immunohistochemistry

A monolayer of iPSC-CMs was fixed with 4% paraformaldehyde for 15 min, incubated in permeabilization solution (1% saponin and 0.05% sodium azide in PBS) for 15 min, and blocked with blocking solution (3% BSA and 0.05% sodium azide in PBS) for 30 min at room temperature. Cells were incubated in primary antibodies against connexin-43 (Abcam, ab11370, 1:1000) and α-actinin (Sigma-Aldrich, A7811, 1:800) for 1 h at room temperature, then washed three times for 2 min with PBS. Secondary antibodies [goat anti-mouse IgG (H+L) cross-adsorbed Alexa Fluor 488 (Thermo Fisher Scientific, A11001) and goat anti-rabbit IgG (H+L) cross-adsorbed Alexa Fluor 594 (Thermo Fisher Scientific, A11012)] were diluted 1:250 in blocking solution and added to the cells to incubate for 1 h at room temperature. Cells were washed with 1× PBS twice for 2 min and incubated with NucBlue Fixed Cell ReadyProbes Reagent (DAPI) (Thermo Fisher Scientific) in PBS for 5 min at room temperature. Coverslips were mounted with ProLong Gold Antifade mountant (Thermo Fisher Scientific) and imaged with a Leica SP8 confocal microscope (Leica, Wetzlar, Germany). Fluorescence intensity was compared using Fiji (ImageJ) software (National Institutes of Health, Bethesda, MD, USA). Cell areas were isolated from the background and mean corrected fluorescence was calculated per cell in each image.

### Flow cytometry

The purity of 45-day-old iPSC-CMs was determined by analysis of cardiomyocyte and fibroblast marker expression by flow cytometry. Single cells were fixed with 2% paraformaldehyde in PBS for 10 min at room temperature; then, 250,000 cells (per parameter tested) were resuspended in 1% BSA in PBS. After fixation, cells were centrifuged at 500 ***g*** for 5 min and resuspended in 100 µl of FACS buffer (0.1% Triton X-100, 1% BSA and 4% goat serum in PBS) supplemented with antibodies and incubated for 45 min at room temperature. The antibodies used are as follows: FITC anti-human CD90 (BioLegend, 328107, 1:20), anti-human α-actinin conjugated with Vio R667 (Miltenyi Biotec, 130-128-591, 1:100) and FITC mouse IgG1 κ Isotype Ctrl (FC) (BioLegend, 400109, 1:20). 900 µl of FACS buffer was added and the cells were centrifuged at 750 ***g*** for 5 min. The pellet was resuspended in 300 µl of 1% BSA in PBS. 1 µl DAPI (Sigma-Aldrich, D9542) was added to cell suspensions. Cells were analysed using a BD LSR II SORP flow cytometer (Becton Dickinson, NJ, USA). A minimum of 10,000 events were recorded and analysed on FlowJo (Becton Dickinson).

### Data analysis and statistical tests

Data analysis, plotting and statistical tests were performed using Microsoft Excel (Microsoft Office 2016, Microsoft, WA, USA), and GraphPad Prism v9 (GraphPad Software, CA, USA). Data were analysed using a mixed effect model using the ‘glmfit’ function in MATLAB, with cell line identity as a fixed categorical variable, and individual differentiations (*N*) and technical replicates (*n*) as random variables. Estimated marginal means were derived from the generalised linear mixed-effect models using the ‘emmeans’ package (https://github.com/jackatta/estimated-marginal-means), with comparison between differentiations undertaken using a Wald test on input contrasts. In all cases, significance was determined with *P*<0.05. Violin SuperPlots of data were created using Violin SuperPlots in MATLAB (Kenny and Schoen, 2021); error bars on violin plots represent estimated marginal means of *N* from the generalised linear model and their standard errors.

## Supplementary Material

10.1242/dmm.050407_sup1 Supplementary information

## References

[DMM050407C1] Antzelevitch, C. (2007). Role of spatial dispersion of repolarization in inherited and acquired sudden cardiac death syndromes. *Am. J. Physiol. Hear. Circ. Physiol.* 293, H2024-H2038. 10.1152/ajpheart.00355.2007 PMC2085107 17586620

[DMM050407C2] Aryana, A., d'Avila, A., Heist, E. K., Mela, T., Singh, J. P., Ruskin, J. N. and Reddy, V. Y. (2007). Remote magnetic navigation to guide endocardial and epicardial catheter mapping of scar-related ventricular tachycardia. *Circulation* 115, 1191-1200. 10.1161/CIRCULATIONAHA.106.672162 17296855

[DMM050407C3] Benjamini, Y., Krieger, A. M. and Yekutieli, D. (2006). Adaptive linear step-up procedures that control the false discovery rate. *Biometrika* 93, 491-507. 10.1093/biomet/93.3.491

[DMM050407C4] Boersma, L., Zetelaki, Z., Brugada, J. and Allessie, M. (2002). Polymorphic reentrant ventricular tachycardia in the isolated rabbit heart studied by high-density mapping. *Circulation* 105, 3053-3061. 10.1161/01.CIR.0000019407.35848.AF 12082002

[DMM050407C5] Briggs, L. E., Takeda, M., Cuadra, A. E., Wakimoto, H., Marks, M. H., Walker, A. J., Seki, T., Oh, S. P., Lu, J. T., Sumners, C. et al. (2008). Perinatal loss of Nkx2-5 results in rapid conduction and contraction defects. *Circ. Res.* 103, 580-590. 10.1161/CIRCRESAHA.108.171835 18689573 PMC2590500

[DMM050407C6] Brundel, B. J. J. M., Van Gelder, I. C., Henning, R. H., Tuinenburg, A. E., Wietses, M., Grandjean, J. G., Wilde, A. A. M., Van Gilst, W. H. and Crijns, H. J. G. M. (2001). Alterations in potassium channel gene expression in atria of patients with persistent and paroxysmal atrial fibrillation: differential regulation of protein and mRNA levels for K+ channels. *J. Am. Coll. Cardiol.* 37, 926-932. 10.1016/S0735-1097(00)01195-5 11693772

[DMM050407C7] Chiswell, K., Zaininger, L. and Semsarian, C. (2023). Evolution of genetic testing and gene therapy in hypertrophic cardiomyopathy. *Prog. Cardiovasc. Dis*. 80, 38-45. 10.1016/j.pcad.2023.04.009 37137376

[DMM050407C8] Clayton, R. H. and Holden, A. V. (2005). Dispersion of cardiac action potential duration and the initiation of re-entry: A computational study. *Biomed. Eng. Online* 4, 11. 10.1186/1475-925X-4-11 15720712 PMC550675

[DMM050407C9] Cohn, R., Thakar, K., Lowe, A., Ladha, F. A., Pettinato, A. M., Romano, R., Meredith, E., Chen, Y.-S., Atamanuk, K., Huey, B. D. et al. (2019). A contraction stress model of hypertrophic cardiomyopathy due to sarcomere mutations. *Stem Cell Rep.* 12, 71-83. 10.1016/j.stemcr.2018.11.015 PMC6335568 30554920

[DMM050407C10] Cortez, D., Schlegel, T. T., Ackerman, M. J. and Bos, J. M. (2017). ECG-derived spatial QRS-T angle is strongly associated with hypertrophic cardiomyopathy. *J. Electrocardiol.* 50, 195-202. 10.1016/j.jelectrocard.2016.10.001 27839835

[DMM050407C61] Cserne Szappanos, H., Viola, H. M., Ito, D. W., Lim, S., Mangala, M., Holliday, M., Barratt Ross, S., Semsarian, C., Hill, A., Dixon, R. E. et al. (2023). Cytoskeletal disarray increases arrhythmogenic vulnerability during sympathetic stimulation in a model of hypertrophic cardiomyopathy. *Sci. Rep.* 13, 11296. 10.1038/s41598-023-38296-2 37438479 PMC10338442

[DMM050407C11] Dainis, A., Zaleta-Rivera, K., Ribeiro, A., Chang, A. C. H., Shang, C., Lan, F., Burridge, P. W., Liu, W. R., Wu, J. C., Chang, A. C. Y. et al. (2020). Silencing of MYH7 ameliorates disease phenotypes in human iPSC-cardiomyocytes. *Physiol. Genomics* 52, 293-303. 10.1152/physiolgenomics.00021.2020 32567507 PMC7468691

[DMM050407C12] Dewenter, M., von der Lieth, A., Katus, H. A. and Backs, J. (2017). Calcium signaling and transcriptional regulation in cardiomyocytes. *Circ. Res.* 121, 1000-1020. 10.1161/CIRCRESAHA.117.310355 28963192

[DMM050407C13] Dhalla, N. S., Dent, M. R., Tappia, P. S., Sethi, R., Barta, J. and Goyal, R. K. (2006). Subcellular remodeling as a viable target for the treatment of congestive heart failure. *J. Cardiovasc. Pharmacol. Ther.* 11, 31-45. 10.1177/107424840601100103 16703218

[DMM050407C14] Dunnink, A., Stams, T. R. G., Bossu, A., Meijborg, V. M. F., Beekman, J. D. M., Wijers, S. C., De Bakker, J. M. T. and Vos, M. A. (2015). Torsade de pointes arrhythmias arise at the site of maximal heterogeneity of repolarization in the chronic complete atrioventricular block dog. *Europace* 19, 858-865. 10.1093/europace/euw087 28525920

[DMM050407C15] Ellesøe, S. G., Johansen, M. M., Bjerre, J. V., Hjortdal, V. E., Brunak, S. Ã., and Larsen, L. A. (2016). Familial atrial septal defect and sudden cardiac death: identification of a novel NKX2-5 mutation and a review of the literature. *Congenit. Hear. Dis.* 11, 283-290. 10.1111/chd.12317 PMC5019245 26679770

[DMM050407C16] Elliott, D. A., Kirk, E. P., Schaft, D. and Harvey, R. P. (2010). Chapter 9.1 - NK-2 Class Homeodomain Proteins: Conserved Regulators of Cardiogenesis. In *Heart development and regeneration* (ed. N. Rosenthal and R. P. Harvey), pp. 569-597. 10.1016/B978-0-12-381332-9.00026-8

[DMM050407C17] Elliott, P. M., Mital, S., Burke, M. A., Day, S. M., Deswal, A., Elliott, P., Evanovich, L. L., Hung, J., Joglar, J. A., Kantor, P. et al. (2014). 2014 ESC Guidelines on diagnosis and management of hypertrophic cardiomyopathy: the Task Force for the Diagnosis and Management of Hypertrophic Cardiomyopathy of the European Society of Cardiology (ESC). *Eur. Hear. J.* 35, 2733-2779. 10.1093/eurheartj/ehu284 25173338

[DMM050407C18] Fabregat, A., Sidiropoulos, K., Viteri, G., Marin-Garcia, P., Ping, P., Stein, L., D'Eustachio, P. and Kelso, J. (2018). Reactome diagram viewer: data structures and strategies to boost performance. *Bioinformatics* 34, 1208-1214. 10.1093/bioinformatics/btx752 29186351 PMC6030826

[DMM050407C19] Flenner, F., Jungen, C., Küpker, N., Ibel, A., Kruse, M., Koivumäki, J. T., Rinas, A., Zech, A. T. L., Rhoden, A., Wijnker, P. J. M. et al. (2021). Translational investigation of electrophysiology in hypertrophic cardiomyopathy. *J. Mol. Cell. Cardiol.* 157, 77-89. 10.1016/j.yjmcc.2021.04.009 33957110 PMC8320769

[DMM050407C20] Geisterfer-Lowrance, A. A. T., Kass, S., Tanigawa, G., Vosberg, H.-P., McKenna, W., Seidman, C. E. and Seidman, J. G. (1990). A molecular basis for familial hypertrophic cardiomyopathy: A β cardiac myosin heavy chain gene missense mutation. *Cell* 62, 999-1006. 10.1016/0092-8674(90)90274-I 1975517

[DMM050407C21] Geisterfer-Lowrance, A. A. T., Christe, M., Conner, D. A., Ingwall, J. S., Schoen, F. J., Seidman, C. E. and Seidman, J. G. (1996). A mouse model of familial hypertrophic cardiomyopathy. *Science* 272, 731-734. 10.1126/science.272.5262.731 8614836

[DMM050407C22] Gough, W. B., Mehra, R., Restivo, M., Zeiler, R. H. and el-Sherif, N. (2018). Reentrant ventricular arrhythmias in the late myocardial infarction period in the dog. 13. Correlation of activation and refractory maps. *Circ. Res.* 57, 432-442. 10.1161/01.RES.57.3.432 4028346

[DMM050407C23] Han, J. and Moe, G. K. (1964). Nonuniform recovery of excitability in ventricular muscle. *Circ. Res.* 14, 44-60. 10.1161/01.RES.14.1.44 14104163

[DMM050407C24] Han, L., Li, Y., Tchao, J., Kaplan, A. D., Lin, B., Li, Y., Mich-Basso, J., Lis, A., Hassan, N., London, B. et al. (2014). Study familial hypertrophic cardiomyopathy using patient-specific induced pluripotent stem cells. *Cardiovasc. Res.* 104, 258-269. 10.1093/cvr/cvu205 25209314 PMC4217687

[DMM050407C25] Helms, A. S., Alvarado, F. J., Yob, J., Tang, V. T., Pagani, F., Russell, M. W., Valdivia, H. H. and Day, S. M. (2016). Genotype-dependent and -independent calcium signaling dysregulation in human hypertrophic cardiomyopathy. *Circulation* 134, 1738-1748. 10.1161/CIRCULATIONAHA.115.020086 27688314 PMC5127749

[DMM050407C26] Holliday, M., Ross, S. B., Lim, S., Mangala, M., Hill, A., Szappanos, H. C., Hool, L. and Semsarian, C. (2018). Development of induced pluripotent stem cells from a patient with hypertrophic cardiomyopathy who carries the pathogenic myosin heavy chain 7 mutation p.Arg403Gln. *Stem Cell Res.* 33, 269-273. 10.1016/j.scr.2018.11.011 30508693

[DMM050407C27] Hueneke, R., Adenwala, A., Mellor, R. L., Seidman, J. G., Seidman, C. E. and Nerbonne, J. M. (2017). Early remodeling of repolarizing K+ currents in the αMHC403/+ mouse model of familial hypertrophic cardiomyopathy. *J. Mol. Cell. Cardiol.* 103, 93-101. 10.1016/j.yjmcc.2017.01.006 28089740 PMC5398411

[DMM050407C28] Ichiki, T., Boerrigter, G., Huntley, B. K., Sangaralingham, S. J., McKie, P. M., Harty, G. J., Harders, G. E. and Burnett, J. C. (2013). Differential expression of the pro-natriuretic peptide convertases corin and furin in experimental heart failure and atrial fibrosis. *Am. J. Physiol. Regul. Integr. Comp. Physiol.* 304, R102-R109. 10.1152/ajpregu.00233.2012 23152112 PMC3543660

[DMM050407C29] Janse, M. J. and Wit, A. L. (1989). Electrophysiological mechanisms of ventricular arrhythmias resulting from myocardial ischemia and infarction. *Physiol. Rev.* 69, 1049-1169. 10.1152/physrev.1989.69.4.1049 2678165

[DMM050407C30] Joyner, R. W. (1986). Modulation of repolarization by electrotonic interactions. *Jpn. Heart J.* 27 Suppl. 1, 167-183. 3820585

[DMM050407C31] Kenny, M. and Schoen, I. (2021). Violin SuperPlots: visualizing replicate heterogeneity in large data sets. *Mol. Biol. Cell* 32, 1333-1334. 10.1091/mbc.E21-03-0130 34264756 PMC8694042

[DMM050407C32] Khoury, E. E., Fokra, A., Kinaneh, S., Knaney, Y., Aronson, D. and Abassi, Z. (2021). Distribution of cardiac and renal corin and proprotein convertase subtilisin/kexin-6 in the experimental model of cardio-renal syndrome of various severities. *Front. Physiol.* 12, 673497. 10.3389/fphys.2021.673497 34733169 PMC8558519

[DMM050407C33] King, J. H., Huang, C. L.-H. and Fraser, J. A. (2013). Determinants of myocardial conduction velocity: implications for arrhythmogenesis. *Front. Physiol.* 4, 154. 10.3389/fphys.2013.00154 23825462 PMC3695374

[DMM050407C34] Kostin, S., Dammer, S., Hein, S., Klovekorn, W. P., Bauer, E. P. and Schaper, J. (2004). Connexin 43 expression and distribution in compensated and decompensated cardiac hypertrophy in patients with aortic stenosis. *Cardiovasc. Res.* 62, 426-436. 10.1016/j.cardiores.2003.12.010 15094362

[DMM050407C35] Lachaud, Q., Aziz, M. H. N., Burton, F. L., Macquaide, N., Myles, R. C., Simitev, R. D. and Smith, G. L. (2022). Electrophysiological heterogeneity in large populations of rabbit ventricular cardiomyocytes. *Cardiovasc. Res.* 118, 3112-3125. 10.1093/cvr/cvab375 35020837 PMC9732512

[DMM050407C36] Lan, F., Lee, A. S., Liang, P., Sanchez-Freire, V., Nguyen, P. K., Wang, L., Han, L., Yen, M., Wang, Y., Sun, N. et al. (2013). Abnormal calcium handling properties underlie familial hypertrophic cardiomyopathy pathology in patient-specific induced pluripotent stem cells. *Cell Stem Cell* 12, 101-113. 10.1016/j.stem.2012.10.010 23290139 PMC3638033

[DMM050407C37] Langenickel, T. H., Pagel, I., Buttgereit, J., Tenner, K., Lindner, M., Dietz, R., Willenbrock, R. and Bader, M. (2004). Rat corin gene: molecular cloning and reduced expression in experimental heart failure. *Am. J. Physiol. Hear. Circ. Physiol.* 287, H1516-H1521. 10.1152/ajpheart.00947.2003 15155264

[DMM050407C38] Lesh, M. D., Pring, M. and Spear, J. F. (2018). Cellular uncoupling can unmask dispersion of action potential duration in ventricular myocardium. A computer modeling study. *Circ. Res.* 65, 1426-1440. 10.1161/01.RES.65.5.1426 2805251

[DMM050407C39] Liu, Y., Beyer, A. and Aebersold, R. (2016). On the dependency of cellular protein levels on mRNA abundance. *Cell* 165, 535-550. 10.1016/j.cell.2016.03.014 27104977

[DMM050407C40] Lyon, A., Bueno-Orovio, A., Zacur, E., Ariga, R., Grau, V., Neubauer, S., Watkins, H., Rodriguez, B. and Mincholé, A. (2018). Electrocardiogram phenotypes in hypertrophic cardiomyopathy caused by distinct mechanisms: apico-basal repolarization gradients vs. Purkinje-myocardial coupling abnormalities. *Europace* 20, iii102-iii112. 10.1093/europace/euy226 30476051 PMC6251182

[DMM050407C41] Magrì, D., Santolamazza, C., Limite, L., Mastromarino, V., Casenghi, M., Orlando, P., Pagannone, E., Musumeci, M. B., Maruotti, A., Ricotta, A. et al. (2017). QT spatial dispersion and sudden cardiac death in hypertrophic cardiomyopathy: Time for reappraisal. *J. Cardiol* 70, 310-315. 10.1016/j.jjcc.2017.01.006 28341542

[DMM050407C42] McIntosh, M. A., Cobbe, S. M. and Smith, G. L. (2000). Heterogeneous changes in action potential and intracellular Ca2+ in left ventricular myocyte sub-types from rabbits with heart failure. *Cardiovasc. Res.* 45, 397-409. 10.1016/S0008-6363(99)00360-0 10728360

[DMM050407C43] Mills, R. J., Lee, I., Hou, C., Weng, C.-C., Li, S., Lieberman, B. P., Zeng, C., Mankoff, D. A. and Mach, R. H. (2017). Functional screening in human cardiac organoids reveals a metabolic mechanism for cardiomyocyte cell cycle arrest. *Proc. Natl. Acad. Sci. USA* 114, E8372-E8381. 10.1073/pnas.1703109114 28916735 PMC5635889

[DMM050407C44] Mines, G. R. (1913). On dynamic equilibrium in the heart. *J. Physiol.* 46, 349-383. 10.1113/jphysiol.1913.sp001596 16993210 PMC1420430

[DMM050407C45] Mosqueira, D., Smith, J. G. W., Bhagwan, J. R. and Denning, C. (2019). Modeling hypertrophic cardiomyopathy: mechanistic insights and pharmacological intervention. *Trends Mol. Med.* 25, 775-790. 10.1016/j.molmed.2019.06.005 31324451

[DMM050407C46] Nakashima, Y., Ono, K., Yoshida, Y., Kojima, Y., Kita, T., Tanaka, M. and Kimura, T. (2009). The search for Nkx2–5-regulated genes using purified embryonic stem cell-derived cardiomyocytes with Nkx2–5 gene targeting. *Biochem. Bioph. Res. Co.* 390, 821-826. 10.1016/j.bbrc.2009.10.056 19836354

[DMM050407C47] Nearing, B. D., Wellenius, G. A., Mittleman, M. A., Josephson, M. E., Burger, A. J. and Verrier, R. L. (2012). Crescendo in depolarization and repolarization heterogeneity heralds development of ventricular tachycardia in hospitalized patients with decompensated heart failure. *Circ. Arrhythm. Electrophysiol.* 5, 84-90. 10.1161/CIRCEP.111.965434 22157521 PMC3296063

[DMM050407C48] Ommen, S. R. and Semsarian, C. (2021). Hypertrophic cardiomyopathy: a practical approach to guideline directed management. *Lancet* 398, 2102-2108. 10.1016/S0140-6736(21)01205-8 34600606

[DMM050407C49] Ommen, S. R., Mital, S., Burke, M. A., Day, S. M., Deswal, A., Elliott, P., Evanovich, L. L., Hung, J., Joglar, J. A., Kantor, P. et al. (2020). 2020 AHA/ACC Guideline for the diagnosis and treatment of patients with hypertrophic cardiomyopathy: executive summary a report of the american college of cardiology/american heart association joint committee on clinical practice guidelines. *J. Am. Coll. Cardiol* 76, 3022-3055. 10.1016/j.jacc.2020.08.044 33229115

[DMM050407C50] Pan, J., Hinzmann, B., Yan, W., Wu, F., Morser, J. and Wu, Q. (2002). Genomic structures of the human and murine corin genes and functional GATA elements in their promoters*. *J. Biol. Chem.* 277, 38390-38398. 10.1074/jbc.M205686200 12154094

[DMM050407C51] Qiao, Y., Lipovsky, C., Hicks, S., Bhatnagar, S., Li, G., Khandekar, A., Guzy, R., Woo, K. V., Nichols, C. G., Efimov, I. R. et al. (2017). Transient notch activation induces long-term gene expression changes leading to sick sinus syndrome in Mice. *Circ. Res.* 121, 549-563. 10.1161/CIRCRESAHA.116.310396 28674041 PMC5565396

[DMM050407C52] Reaume, A. G., de Sousa, P. A., Kulkarni, S., Langille, B. L., Zhu, D., Davies, T. C., Juneja, S. C., Kidder, G. M. and Rossant, J. (1995). Cardiac malformation in neonatal mice lacking connexin43. *Science* 267, 1831-1834. 10.1126/science.7892609 7892609

[DMM050407C53] Robinson, P., Liu, X., Sparrow, A., Patel, S., Zhang, Y.-H., Casadei, B., Watkins, H. and Redwood, C. (2018). Hypertrophic cardiomyopathy mutations increase myofilament Ca2+ buffering, alter intracellular Ca2+ handling, and stimulate Ca2+-dependent signaling. *J Biological Chem* 293, 10487-10499. 10.1074/jbc.RA118.002081 PMC6036197 29760186

[DMM050407C54] Saumarez, R. C. (1994). Electrophysiological investigation of patients with hypertrophic cardiomyopathy: Evidence that slowed intraventricular conduction is associated with an increased risk of sudden death. *Heart* 72, S19-S23. 10.1136/hrt.72.6_Suppl.S19 PMC1025671 7873318

[DMM050407C55] Saumarez, R. C., Camm, A. J., Panagos, A., Gill, J. S., Stewart, J. T., de Belder, M. A., Simpson, I. A. and McKenna, W. J. (2018). Ventricular fibrillation in hypertrophic cardiomyopathy is associated with increased fractionation of paced right ventricular electrograms. *Circulation* 86, 467-474. 10.1161/01.CIR.86.2.467 1638716

[DMM050407C56] Schneider, C. A., Rasband, W. S. and Eliceiri, K. W. (2012). NIH Image to ImageJ: 25 years of image analysis. *Nat. Methods* 9, 671-675. 10.1038/nmeth.2089 22930834 PMC5554542

[DMM050407C57] Schumacher, B., Gietzen, F. H., Neuser, H., Schümmelfeder, J., Schneider, M., Kerber, S., Schimpf, R., Wolpert, C. and Borggrefe, M. (2005). Electrophysiological characteristics of septal hypertrophy in patients with hypertrophic obstructive cardiomyopathy and moderate to severe symptoms. *Circulation* 112, 2096-2101. 10.1161/CIRCULATIONAHA.104.515643 16186424

[DMM050407C58] Semsarian, C., Healey, M. J., Fatkin, D., Giewat, M., Duffy, C., Seidman, C. E. and Seidman, J. G. (2001). A polymorphic modifier gene alters the hypertrophic response in a murine model of familial hypertrophic cardiomyopathy. *J. Mol. Cell. Cardiol* 33, 2055-2060. 10.1006/jmcc.2001.1466 11708849

[DMM050407C59] Semsarian, C., Ahmad, I., Giewat, M., Georgakopoulos, D., Schmitt, J. P., McConnell, B. K., Reiken, S., Mende, U., Marks, A. R., Kass, D. A. et al. (2002). The L-type calcium channel inhibitor diltiazem prevents cardiomyopathy in a mouse model. *J. Clin. Investig.* 109, 1013-1020. 10.1172/JCI200214677 11956238 PMC150949

[DMM050407C60] Semsarian, C., Ingles, J., Maron, M. S. and Maron, B. J. (2015). New Perspectives on the Prevalence of Hypertrophic Cardiomyopathy. *J. Am. Coll. Cardiol.* 65, 1249-1254. 10.1016/j.jacc.2015.01.019 25814232

[DMM050407C62] Tan, A. Y., Nearing, B. D., Rosenberg, M., Nezafat, R., Josephson, M. E. and Verrier, R. L. (2017). Interlead heterogeneity of R- and T-wave morphology in standard 12-lead ECGs predicts sustained ventricular tachycardia/fibrillation and arrhythmic death in patients with cardiomyopathy. *J. Cardiovasc. Electrophysiol* 28, 1324-1333. 10.1111/jce.13288 28675579

[DMM050407C63] Toepfer, C. N., Garfinkel, A. C., Venturini, G., Wakimoto, H., Repetti, G., Alamo, L., Sharma, A., Agarwal, R., Ewoldt, J. K., Cloonan, P. et al. (2019). Myosin sequestration regulates sarcomere function, cardiomyocyte energetics, and metabolism, informing the pathogenesis of hypertrophic cardiomyopathy. *Circulation* 141, 828-842. 10.1161/CIRCULATIONAHA.119.042339 PMC7077965 31983222

[DMM050407C64] Tran, K. L., Lu, X., Lei, M., Feng, Q. and Wu, Q. (2004). Upregulation of corin gene expression in hypertrophic cardiomyocytes and failing myocardium. *Am. J. Physiol Heart C* 287, H1625-H1631. 10.1152/ajpheart.00298.2004 15191894

[DMM050407C65] van Rijen, H. V. M., Eckardt, D., Degen, J., Theis, M., Ott, T., Willecke, K., Jongsma, H. J., Opthof, T. and de Bakker, J. M. T. (2004). Slow conduction and enhanced anisotropy increase the propensity for ventricular tachyarrhythmias in adult mice with induced deletion of connexin43. *Circulation* 109, 1048-1055. 10.1161/01.CIR.0000117402.70689.75 14967725

[DMM050407C66] Varnava, A. M., Elliott, P. M., Mahon, N., Davies, M. J. and McKenna, W. J. (2001). Relation between myocyte disarray and outcome in hypertrophic cardiomyopathy. *Am. J. Cardiol.* 88, 275-279. 10.1016/S0002-9149(01)01640-X 11472707

[DMM050407C67] Viola, H. M. and Hool, L. C. (2019). Impaired calcium handling and mitochondrial metabolic dysfunction as early markers of hypertrophic cardiomyopathy. *Arch. Biochem. Biophys.* 665, 166-174. 10.1016/j.abb.2019.03.006 30885674

[DMM050407C68] Wolf, C. M., Moskowitz, I. P. G., Arno, S., Branco, D. M., Semsarian, C., Bernstein, S. A., Peterson, M., Maida, M., Morley, G. E., Fishman, G. et al. (2005). Somatic events modify hypertrophic cardiomyopathy pathology and link hypertrophy to arrhythmia. *Proc. Natl. Acad. Sci. USA* 102, 18123-18128. 10.1073/pnas.0509145102 16332958 PMC1307513

[DMM050407C69] Writing Committee Members, Ommen, S. R., Mital, S., Burke, M. A., Day, S. M., Deswal, A., Elliott, P., Evanovich, L. L., Hung, J., Joglar, J. A. et al. (2020). 2020 AHA/ACC Guideline for the diagnosis and treatment of patients with hypertrophic cardiomyopathy a report of the American college of cardiology/American heart association joint committee on clinical practice guidelines. *J. Am. Coll. Cardiol* 76, e159-e240. 10.1016/j.jacc.2020.08.045 33229116

[DMM050407C70] Wu, H., Yang, H., Rhee, J.-W., Zhang, J. Z., Lam, C. K., Sallam, K., Chang, A. C. Y., Ma, N., Lee, J., Zhang, H. et al. (2019). Modelling diastolic dysfunction in induced pluripotent stem cell-derived cardiomyocytes from hypertrophic cardiomyopathy patients. *Eur. Hear. J.* 40, 3685-3695. 10.1093/eurheartj/ehz326 PMC7963137 31219556

[DMM050407C71] Zahid, S., Cochet, H., Boyle, P. M., Schwarz, E. L., Whyte, K. N., Vigmond, E. J., Dubois, R., Hocini, M., Haïssaguerre, M., Jaïs, P. et al. (2016). Patient-derived models link re-entrant driver localization in atrial fibrillation to fibrosis spatial pattern. *Cardiovasc. Res.* 110, 443-454. 10.1093/cvr/cvw073 27056895 PMC4872878

[DMM050407C72] Zhang, Y., Wang, H., Kovacs, A., Kanter, E. M. and Yamada, K. A. (2009). Reduced expression of Cx43 attenuates ventricular remodeling after myocardial infarction via impaired TGF-beta signaling. *Am. J. Physiol. Hear. Circ. Physiol.* 298, H477-H487. 10.1152/ajpheart.00806.2009 PMC2822575 19966054

[DMM050407C73] Zhang, J. Z., Termglinchan, V., Shao, N.-Y., Itzhaki, I., Liu, C., Ma, N., Tian, L., Wang, V. Y., Chang, A. C. Y., Guo, H. et al. (2019). A human iPSC double-reporter system enables purification of cardiac lineage subpopulations with distinct function and drug response profiles. *Cell Stem Cell* 24, 802-811.e5. 10.1016/j.stem.2019.02.015 30880024 PMC6499654

