# Supplementary material for: Reduced connexin-43 expression, slow conduction and repolarisation dispersion in a model of hypertrophic cardiomyopathy
Source: Dis Model Mech. 2024 Aug 27;17(8):dmm050407. doi: 10.1242/dmm.050407 (PMC11381919; doi:10.1242/dmm.050407)
Supplement: Supplementary information [file dmm-17-050407-s1.pdf]

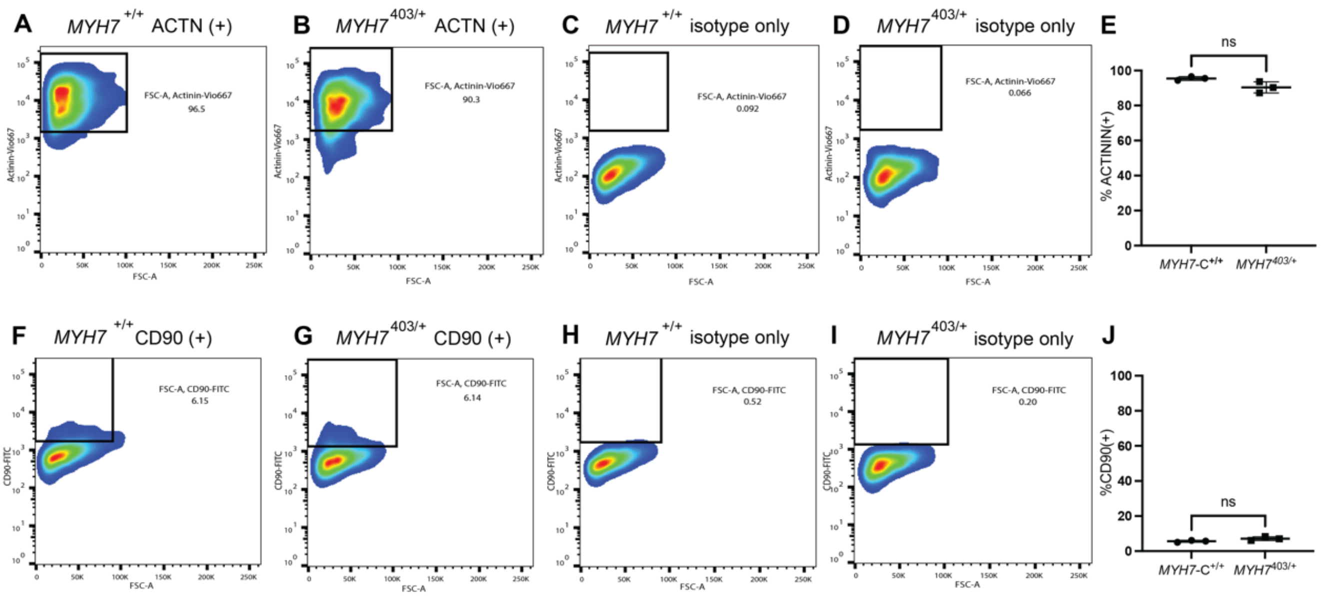

Fig. S1. Flow cytometry assessment of cardiomyocyte purity. A/B) Representative flow cytometry gating for ACTN positive MYH7-C$^{+/+}$ and MYH7$^{403/+}$iPSC-CMs. C/D) Representative gating for unstained controls for MYH7-C$^{+/+}$ (0.05% ± 0.03) and MYH7$^{403/+}$ (0.12% ± 0.07). E) Percentage of ACTN positive iPSC-CMs (MYH7-C$^{+/+}$: 95.47% ± 1.05 and MYH7$^{403/+}$: 90.40% ± 3.15). F/G) Representative flow cytometry gating for CD90 positive iPSC-CMs. H/I) Representative gating for isotype controls for MYH7-C$^{+/+}$ (0.35% ± 0.10) and MYH7$^{403/+}$ (0.12% ± 0.08). J) Percentage of CD90 positive iPSC-CMs (MYH7-C$^{+/+}$: 5.64% ± 0.57) and MYH7$^{403/+}$ (7.15% ± 1.09).

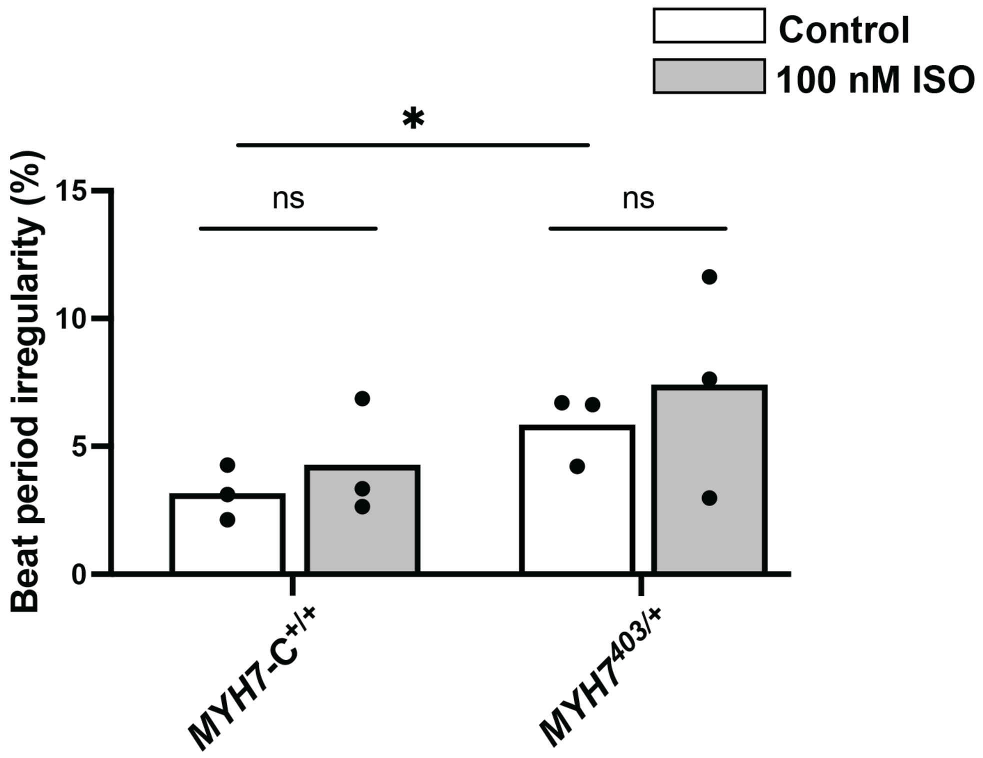

Fig. S2. Effect of β-Adrenergic stimulation on beat period irregularity in MYH7-C$^{+/+}$ and MYH7$^{403/+}$ iPSC-CMs. 100 nM isoproterenol had no significant effect on beat rate irregularity (measured as coefficient of variation in cycle length) in either MYH7-C$^{+/+}$ (N=3 differentiations, n = 16 wells) and MYH7$^{403/+}$ (N=3 differentiation, n=18 wells).

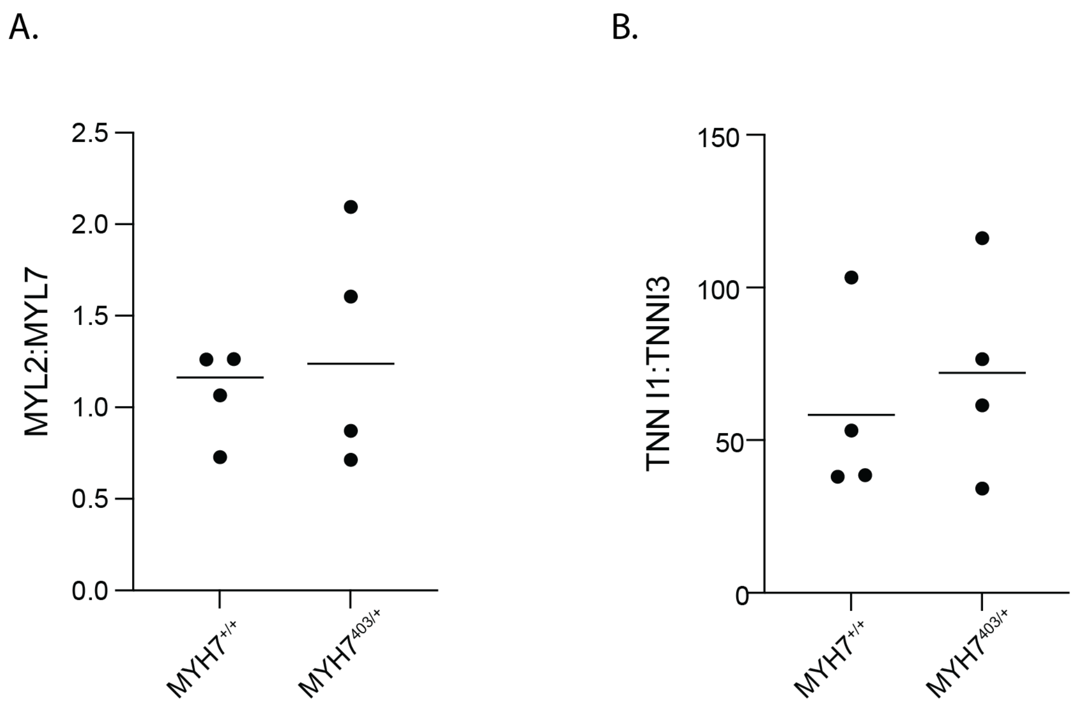

Fig. S3. Expression ratios of sarcomere genes in *MYH7*$^{+/+}$ versus *MYH7*$^{403/+}$.

Disease Models & Mechanisms • Supplementary information

Original, full-sized Western blots for images shown in Fig. 6C

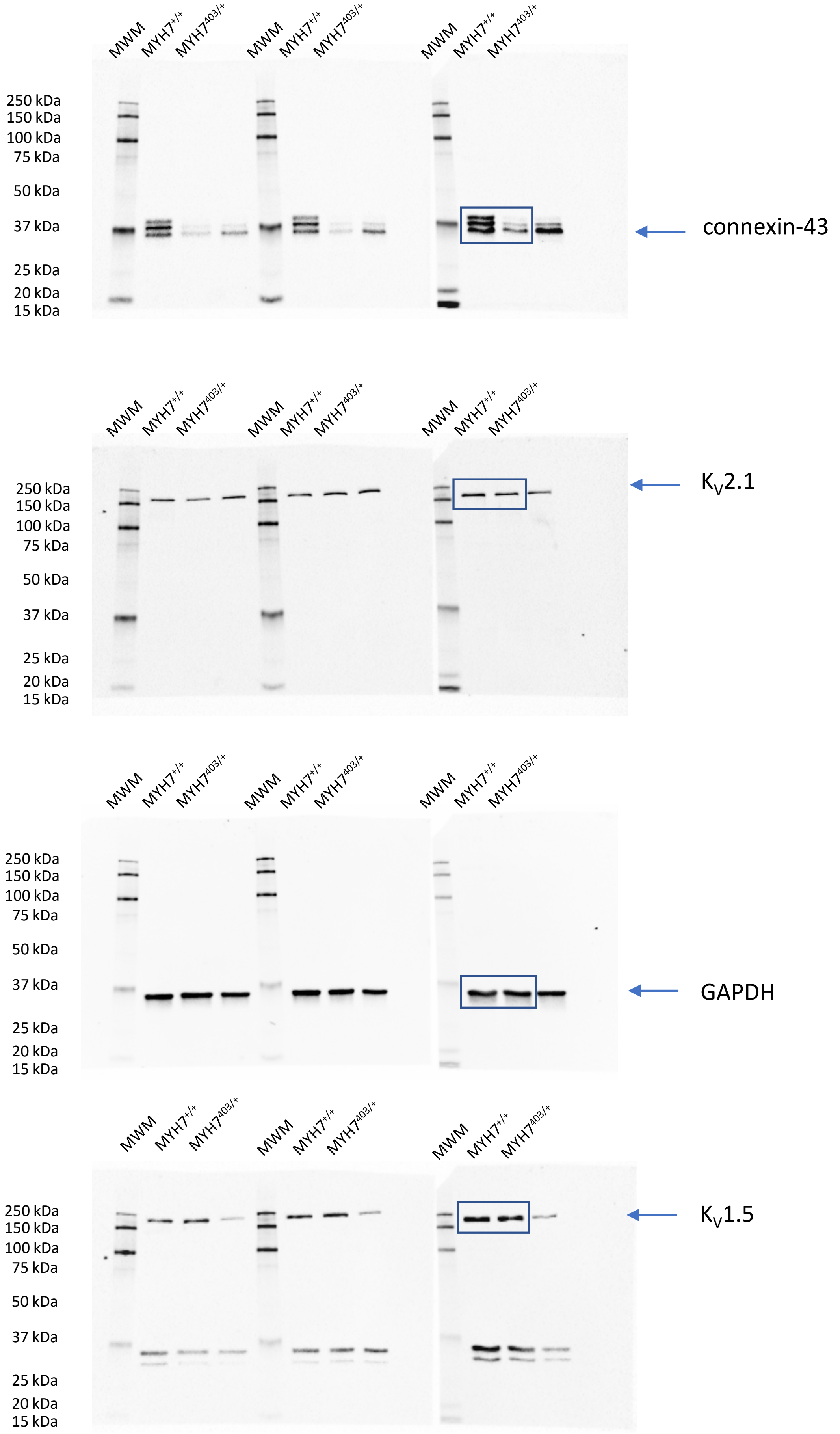

Membrane probed with: rabbit anti-connexin-43; then guinea pig anti-K_V2.1; after stripping it was re-probed with rabbit anti-GAPDH; then rabbit anti-K_V1.5 antibodies

Disease Models & Mechanisms • Supplementary information

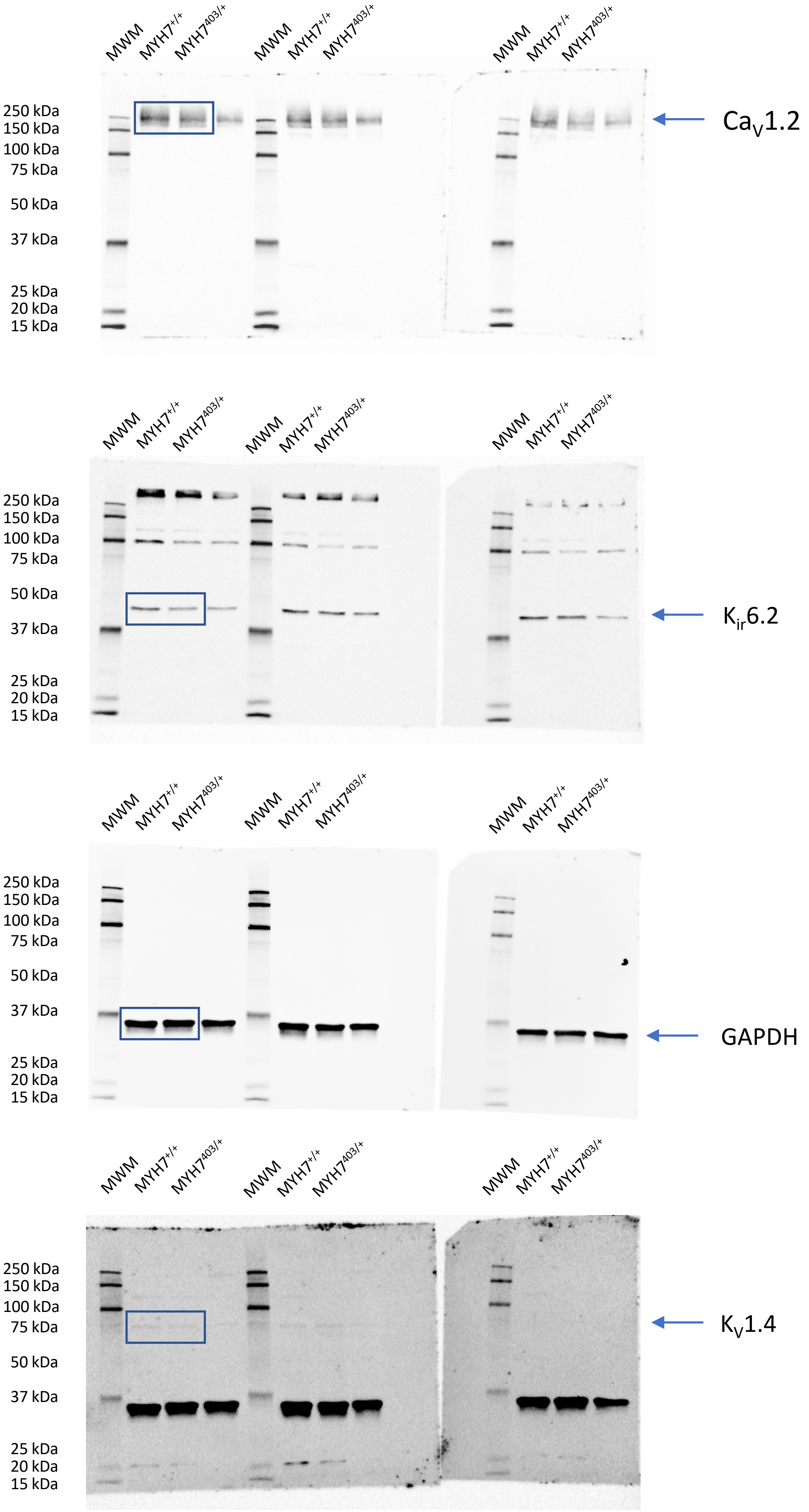

Membrane probed with: rabbit anti-Ca_V1.2; stripped, then re-probed with rabbit anti-K_{ir}6.2; stripped, then re-probed with rabbit anti-GAPDH; then rabbit anti-K_V1.4 antibodies

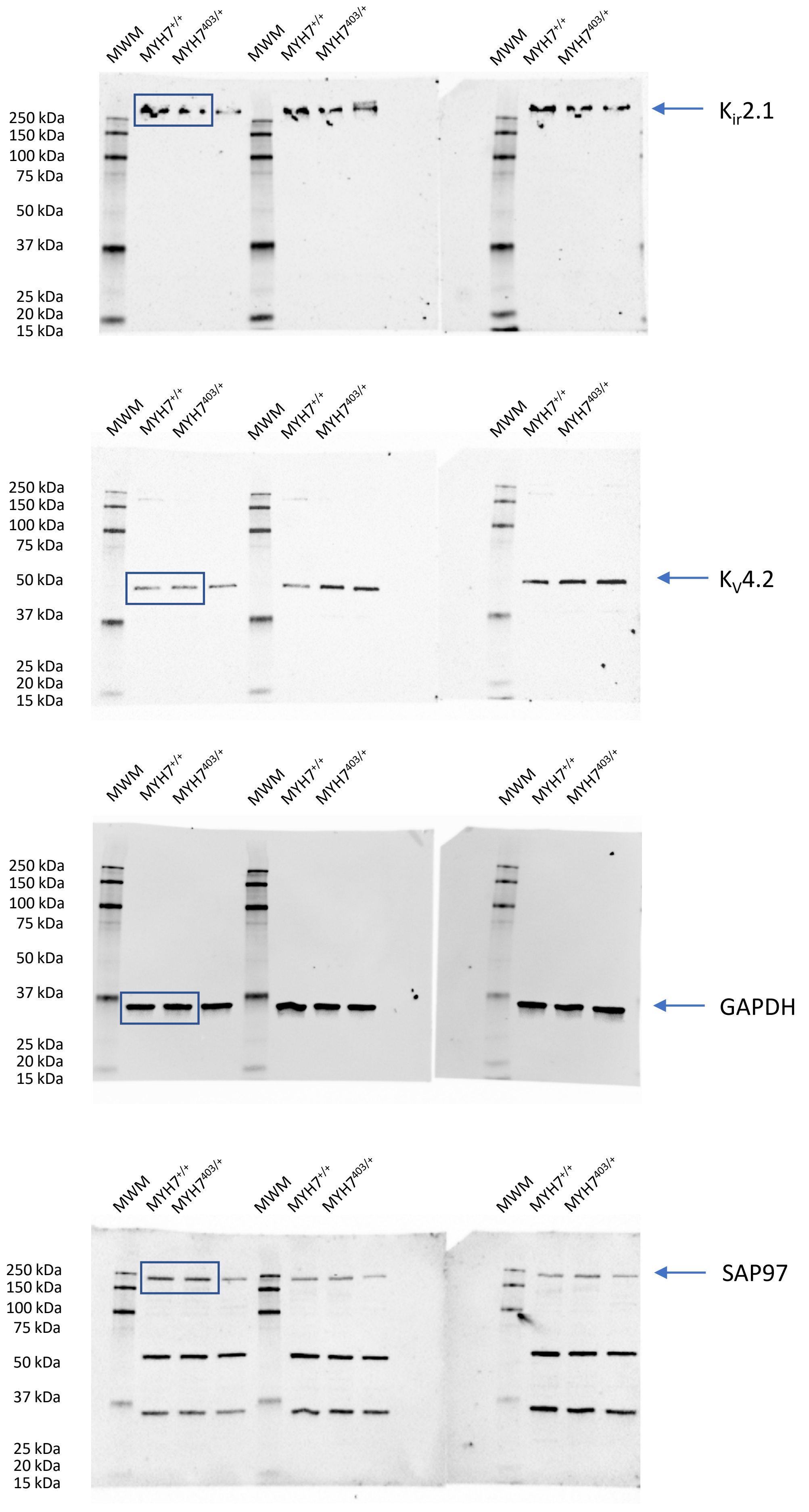

Membrane probed with: guinea pig anti-K_{ir}2.1; then rabbit anti-K_V4.2; stripped then re-probed with rabbit anti-GAPDH; then rabbit anti-SAP97

Disease Models & Mechanisms • Supplementary information

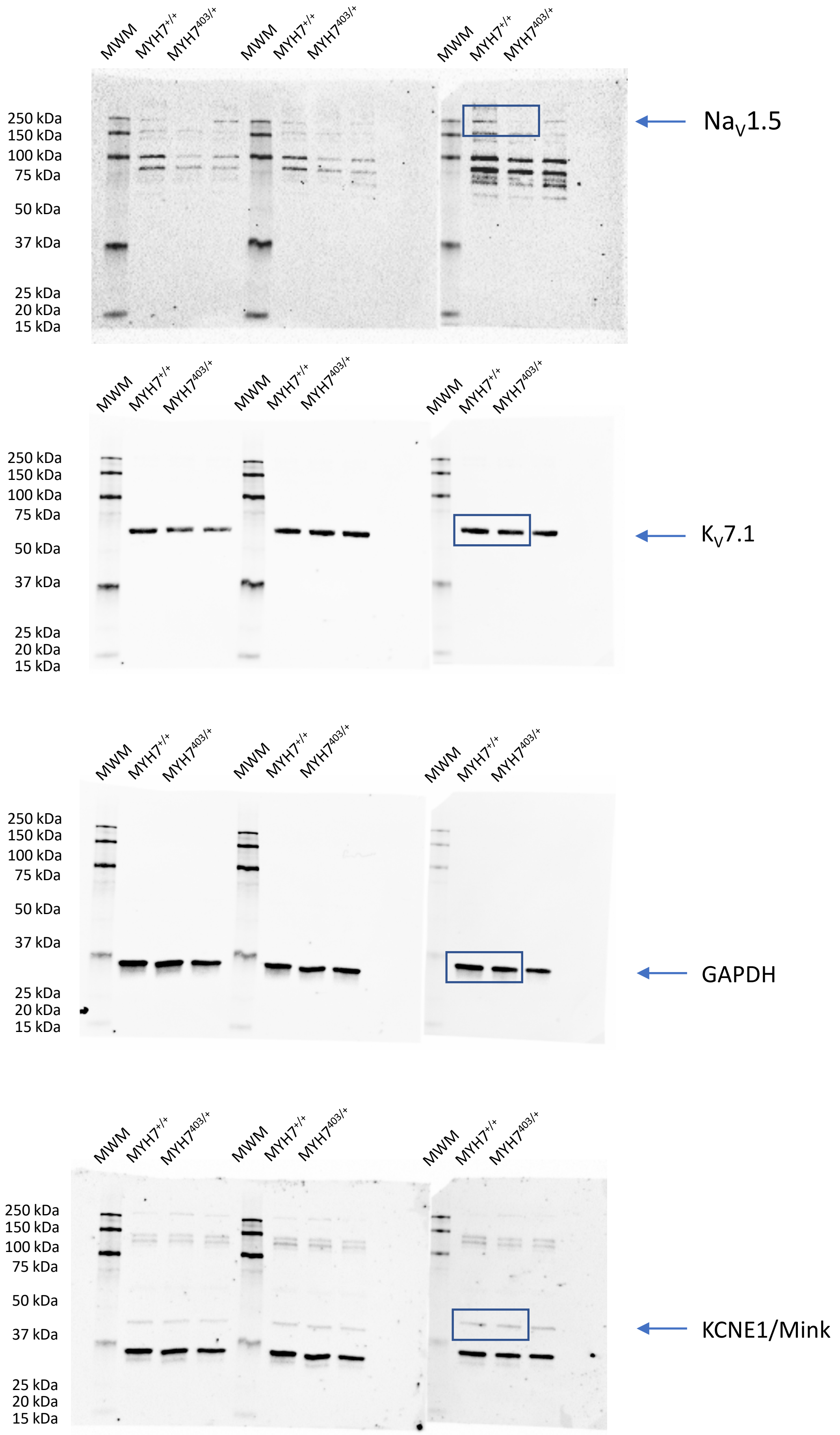

Membrane probed with: rabbit anti-Na_V1.5; stripped then re-probed with rabbit anti-K_V7.1; stripped then re-probed with rabbit anti-GAPDH, stripped then re-probed with rabbit anti-KCNE1/Mink antibodies

Disease Models & Mechanisms • Supplementary information

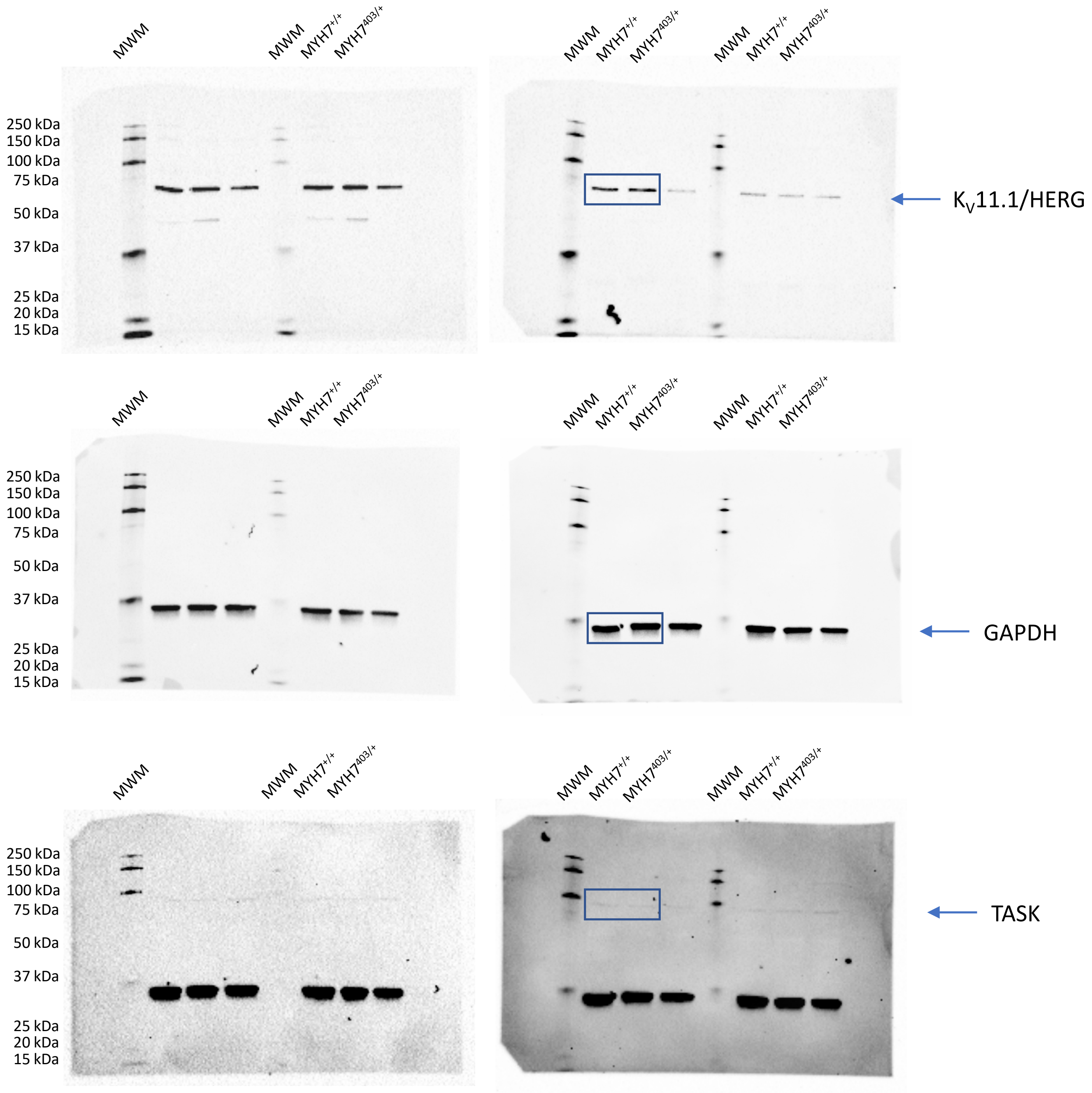

Membrane probed with: rabbit anti-K_V11.1/HERG; stripped and re-probed with rabbit anti-GAPDH; then rabbit anti-TASK antibodies

Fig. S4. Blot transparency data.
